# Inflammatory microglia signals drive A1-like polarization of astrocytes even in the presence of HIV-1 Tat

**DOI:** 10.1007/s12035-025-05409-z

**Published:** 2025-12-04

**Authors:** Jill M. Lawrence, Will Dampier, Joshua Chang Mell, Diehl R. De Souza, Kayla Schardien, Kyle Yeakle, R. Jordan Barnett, Bhaswati Sen, Azad Ahmed, Michael Bouchard, Brian Wigdahl, Michael R. Nonnemacher

**Affiliations:** 1 Molecular and Cell Biology and Genetics Graduate Program, Drexel University College of Medicine, Philadelphia, PA USA; 2 Department of Microbiology and Immunology, Drexel University College of Medicine, 245 N 15 St, Philadelphia, PA 19102 USA; 3 Center for Molecular Virology and Translational Neuroscience, Institute for Molecular Medicine and Infectious Disease, Drexel University College of Medicine, Philadelphia, PA USA; 4 Center for Advanced Microbial Processing, Institute for Molecular Medicine and Infectious Disease, Drexel University College of Medicine, Philadelphia, PA USA; 5 Center for Genomic Sciences, Institute for Molecular Medicine and Infectious Disease, Drexel University College of Medicine, Philadelphia, PA USA; 6 Genomics Core Facility, Institute for Molecular Medicine and Infectious Disease, Drexel University College of Medicine, Philadelphia, PA USA; 7 Department of Neurobiology and Anatomy, Drexel University College of Medicine, 2900 W. Queen Lane, Philadelphia, PA 19129 USA; 8 Department of Biochemistry and Molecular Biology, Drexel University College of Medicine, 245 N 15Th St, Philadelphia, PA 19102 USA; 9 Microbiology and Immunology Graduate Program, Drexel University College of Medicine, Philadelphia, PA USA; 10 Sidney Kimmel Cancer Center, Thomas Jefferson University, Philadelphia, PA USA

**Keywords:** Astrocytes, A1, Neuroinflammation, Co-culture model, Blood–brain barrier, HIV-1 Tat

## Abstract

In the context of neurodegeneration, activated microglia facilitate inflammation via secretion of TNF-α, IL-1α, and C1q. Astrocytes exposed to this signaling array polarize to a reactive inflammatory phenotype, termed A1 or A1-like. Astrocytes are essential for neuronal survival, synaptic support, and blood–brain barrier (BBB) function, but A1-like astrocytes upregulate inflammatory gene expression, downregulate neurotrophic factors, and secrete neurotoxic signals. The consequences of A1-like polarization on BBB function are unknown but may have etiological implications for some diseases. Frequently identified by upregulation of complement component 3 (C3), A1-like astrocytes have been characterized in neurodegenerative disorders like Alzheimer’s disease, with polarization correlated with disease progression and severity. However, the role of A1-like astrocytes in neurodegeneration associated with chronic viral infections, like HIV-1-associated neurocognitive disorder (HAND), remains unclear. An in vitro system using primary human astrocytes, as well as a BBB model featuring primary human brain microvascular endothelial cells (BMECs) co-cultured with astrocytes, was used to elucidate cellular and molecular consequences of chronic astrocyte activation. As measured by whole transcriptome analysis and protein expression assays, repeated treatment with TNF-α, IL-1α, and C1q induced A1-like polarization of astrocytes both in monoculture and in a BBB model, resulting in increased secretion of pro-inflammatory signals. No substantial change to BBB permeability was observed. In contrast, exposure to HIV-1 viral protein Tat did not independently induce A1-like polarization. Ongoing investigations into the effect of astrocyte polarization on BBB integrity and treatment with pathogenic proteins may provide insights into the role of neurotoxic astrocytes in neurovirologic pathologies.

**Supplementary Information:**

The online version contains supplementary material available at 10.1007/s12035-025-05409-z.

## Introduction

Astrocytes play a vital role in maintaining the collective homeostasis of the central nervous system (CNS) by supporting other glial cells, neurons, synapses, and the blood–brain barrier (BBB) [1, 2]. The BBB, or neurovascular unit, is comprised of tightly packed microvascular endothelial cells supported by pericytes and astrocytes, and envelopes cerebral capillaries as an added layer of protection between the CNS and periphery [2, 3]. Tight junctions between the endothelial cells of the BBB establish a low level of permeability, and the incoming blood supply is regulated by dense astrocyte processes forming a charged glial limiting membrane [3–5]. These astrocytic endfeet are lined with junctional proteins and the astrocyte-specific water channel, aquaporin-4 (AQP4) [2, 3, 6]. An important role of the BBB is the restriction of large-scale transmigration of peripheral immune cells. Failure to maintain barrier integrity may permit such infiltrations, resulting in neuroinflammation due to secretion of inflammatory cytokines and reactive oxygen species (ROS) by invading immune cells [2, 7]. In this way, astrocytes supporting the BBB play an important role in preventing neurotoxic insults in the CNS.

In response to damage or injury, quiescent astrocytes become reactive and adopt different functional phenotypes depending on the nature of the inducing stimuli. These phenotypes range from neurotoxic to neuroprotective, with different polarization states having associated changes in gene expression and cellular function. Two of the primary reactive astrocyte phenotypes are often classed as “A1” and “A2” [8, 9]. Astrocytes polarize to a neuroprotective A2 phenotype in response to tissue injury and ischemia. Astrocyte polarization to a neurotoxic A1 phenotype occurs in response to neuroinflammation, aging, and infection. The A1 reactive state is characterized by secretion of neurotoxic factors, neuronal cytotoxicity, reduced support of neurons and synapses via decreased expression of neurotrophic and synaptogenic factors, and increased expression of pro-inflammatory signals [2, 8–11]. More recent research has emphasized that astrocyte phenotypes can be variable and dynamic, rather than strictly adhering to fixed states [2, 12]. The terms “A1-like” and “A2-like” are used to refer to a general pattern of phenotypic changes that can be broadly classified as pro-inflammatory and proliferative, respectively.

Neuroinflammation, and the presence of misfolded, cytotoxic, or pathogenic proteins, activates surveilling microglia that adopt a reactive phagocytic phenotype that expresses various inflammatory mediators, including tumor necrosis factor-α (TNF-α), interleukin-1α (IL-1α), and complement component subunit 1q (C1q) [4, 11]. This combination of signals secreted by pro-inflammatory microglia has been identified as one of the primary mechanisms that initiates the intracellular cascade underlying A1-like polarization. It is important to note that other mechanisms, such as exposure to pathogenic proteins, have also been observed to induce the phenotypic switch [2, 13–15]. Polarization to an A1-like phenotype is associated with activation of the NF-κB pathway and subsequent upregulation of inflammatory gene expression, including complement component 3 (C3). Specifically, ligand binding to cytokine receptors on the surface of astrocytes results in phosphorylation and dissociation of an intracellular complex containing inactive p50 and p65. These released transcription factors are phosphorylated and translocated to the nucleus where they can influence gene expression [2, 10, 16–18]. Upregulation of C3 and other complement cascade genes by neurotoxic astrocytes can result in inflammation, synaptic damage, and neuronal excitotoxicity [10, 19, 20]. C3 in particular binds to a C3 receptor on neurons and disrupts intracellular Ca^2+^ homeostasis [14]. A1-like astrocytes also display increased expression of chemoattractant, particularly CXCL10, a signal that directs T cell migration [10, 11, 21, 22]. Chemoattraction of immune cells further establishes a pro-inflammatory environment in the CNS. A1-like astrocytes secrete several factors with functions that remain poorly understood, including neurotoxic factors yet to be identified [2, 23, 24]. There is some evidence to suggest that A1-like astrocytes exhibit altered expression of AQP4, which may contribute to impaired BBB integrity and overall dysregulation of water homeostasis [25, 26].

A1 or A1-like reactive astrocytes have been identified in aging, along with a variety of neurodegenerative disorders, and their prevalence has been associated with increasing disease severity [2]. A1-like reactive astrocytes have been observed in Alzheimer’s disease, Parkinson’s disease, Huntington’s disease, ALS, and multiple sclerosis, with up to half of astrocytes in diseased brain regions displaying A1-like polarization [9–11]. Some preliminary evidence suggests that A1-like polarization can occur in the context of infectious neuroviral disease, specifically canine distemper demyelinating leukoencephalitis, which is also associated with a corresponding loss of AQP4 expression [25]. In a mouse model of normal aging, astrocytes upregulate Ca^2+^ signaling, thereby disrupting neuronal signaling [27]. Microglia activation is another hallmark of aging. Subsequent TNF-α, IL-1α, and C1q signaling results in an increased proportion of astrocytes polarizing to an A1-like phenotype, particularly among murine hippocampal and striatal astrocytes, a process that may underly age-associated vulnerability and cognitive deficits [9–11, 28]. This age-associated astrocyte polarization facilitated by microglia activation is amplified in the context of simultaneous inflammation [11].

In contrast to the high mortality of human immunodeficiency virus type 1 (HIV-1) infections at the height of the acquired immunodeficiency syndrome (AIDS) epidemic, the advent and increasing availability of combination anti-retroviral therapy (cART) has drastically improved the prognosis for the nearly 40 million people worldwide currently living with a HIV-1 (PLWH) [29, 30]. There is now a rapidly growing demographic of aging PLWH with associated geriatric health complications, and understanding how HIV-1 infection and age intersect is of increasing importance [30]. Of particular interest are neurological complications that frequently arise in PLWH, classified as HIV-1-associated neurocognitive disorder (HAND). As a neurodegenerative disease, HAND symptomology often resembles Alzheimer’s disease, and both age-associated neurodegenerative conditions involve similar subcortical structures [31]. Symptom severity is reduced in virally suppressed cART patients; however, overall prevalence is unaffected, with up to 50% of aviremic HIV patients predicted to develop HAND [32].

HIV-1 infiltrates the CNS soon after initial exposure via infected T lymphocytes and monocytes, facilitating subsequent infection of perivascular macrophages and microglia [33–35]. Neurotoxicity in HIV-1 infection has been linked to chronic extracellular presence of the viral regulatory protein transactivator of transcription (Tat) in the CNS [36, 37]. In addition to HIV-1 Tat translocation into the CNS across the BBB, the viral protein is also continuously secreted by infected CNS cells, including microglia [36, 38, 39]. As its secretion requires successful integration of HIV-1 into the host cell genome, neurotoxic HIV-1 Tat expression is unaffected by cART treatments that work by preventing viral replication [37]. Constitutively expressed HIV-1 Tat can exert pathogenic effects on local CNS cells. Of note is HIV-1 Tat-induced microglia activation, increased expression of TNF-α and IL-1α, neurodegeneration, and potential disruption of BBB integrity [39–43].

Given this, we hypothesized that chronic exposure to even low levels of HIV-1 Tat may stimulate A1-like polarization in local astrocytes. Any subsequent loss of normal astrocyte functions, pro-inflammatory signaling, and potential impairment of BBB integrity may contribute to HAND pathology in HIV-infected patients. Furthermore, the effect of astrocytes polarized to an A1-like phenotype at the BBB has yet to be elucidated [2]. To study these facets of astrocyte reactivity, an in vitro model of pro-inflammatory astrocyte polarization was developed using primary human fetal cells, both in monoculture and in a co-culture BBB model. An array of analyses, from RNA transcriptional profiles to changes in signal secretion, were used to characterize an A1-like phenotype in stimulated primary human astrocytes. Given the overlap in markers between phenotypes, we aimed to use these analyses across different markers to paint a broad picture of the functional phenotype of these cells, rather than provide an over-prescriptive A1-or-A2 classification. This research also investigated if HIV-1 Tat could induce polarization of astrocytes independent of microglia signaling, as previously observed with pathogenic proteins in Alzheimer’s disease and *T. gondii* infection [13–15]. Significant upregulation of A1-associated markers following chronic exposure to HIV-1 Tat was not observed, suggesting that the viral protein did not independently elicit an A1-like polarization response. This model of A1-like primary human astrocyte activation, and its application in an in vitro BBB model, can be applied to a wide range of research endeavors. These findings expand the growing body of work investigating the effects of inflammatory astrocyte polarization, particularly at the BBB, as well as the role of A1-like astrocytes in viral infection-associated neurodegeneration, which may provide insight into HAND etiology and the unique risks PLWH face.

## Methods

### Primary Cell Culture

Primary human fetal astrocytes harvested from 16 to 18-week aborted fetuses were obtained prior to 2018 from the Temple University/Drexel University Comprehensive NeuroHIV Center (CNHC) Core (Philadelphia, PA, USA) in full compliance with National Institutes of Health and Temple University ethical guidelines. Astrocytes were cultured in Dulbecco’s Modified Eagle Medium (DMEM; Thermo-Fisher Scientific, Waltham, MA, USA) supplemented with 10% heat-inactivated fetal bovine serum (FBS; Gibco; Thermo-Fisher Scientific, Waltham, MA, USA) and 1% penicillin–streptomycin (Corning, Glendale, AZ, USA) on 100 mm TPP Tissue Culture dishes (Midwest Scientific, Fenton, MO, USA). Experiments were only performed with astrocytes between passages 2—6. On 6-well plates (Falcon, Corning, Glendale, AZ, USA), astrocytes were seeded at a density of 90,000 cells/plate and grown to functional confluence.

Primary human brain microvascular endothelial cells (BMECs; Cell Systems, Kirkland, WA, USA) were cultured in Medium 199 (M199; with Phenol Red and 25 mM HEPES, Sigma-Aldrich, St. Louis, MO, USA) supplemented with 20% heat-inactivated newborn calf serum (New Zealand origin; Gibco; Thermo-Fisher Scientific, Waltham, MA, USA), 5% heat-inactivated human serum AB (Fisher Scientific, Waltham, MA, USA), 1% penicillin–streptomycin (Corning, Glendale, AZ, USA), 0.8% L-glutamine (200 mM, Thermo-Fisher Scientific, Waltham, MA, USA), 0.25% endothelial cell growth supplement (ECGS, bovine neural tissue, Sigma-Aldrich, St. Louis, MO, USA), 0.1% ascorbic acid (Sigma-Aldrich, St. Louis, MO, USA), 0.1% heparin (Sigma-Aldrich, St. Louis, MO, USA), and 0.05% bovine brain extract (Lonza, Wayne, PA, USA) (M199-C). BMECs were cultured on 100 mm TPP Tissue Culture dishes (Midwest Scientific, Fenton, MO, USA) coated with 0.2% gelatin (type A, Fisher Scientific, Waltham, MA, USA) and experiments were performed with BMECs between passages 4–18.

### In Vitro Blood-Brain Barrier Model 

To model the BBB in vitro, a co-culture transwell system was used that was originally developed by Eugenin and Berman [44] and validated in our laboratory [45, 46]. Briefly, astrocytes were seeded on the underside of inverted PET transwell inserts with a pore size of 3.0 µm (0.3 cm^2^, Falcon, Fisher Scientific, Waltham, MA, USA) at a density of 100,000 cells/well [47–49]. This process involved feeding astrocytes every 5 to 45 min for 4 h. Following this basal seeding procedure, the transwell inserts were inverted and placed within a 24-well plate (Falcon, Fisher Scientific, Waltham, MA, USA), and BMECs were seeded on the 0.2% gelatin-coated apical side of the transwell membrane at a density of 40,000 cells/well. Both the upper and lower chambers were then incubated with M199-C media under 5% CO_2_ at 37°C. On day 3 of co-culture, the transwell inserts were transferred from M199-C to a low-serum M199-C media that lacked supplementation with human serum and ECGS and included only half of the standard amount of newborn calf serum. Low-serum media was used during the experimental trial to avoid any undesired stimulation or interference with cytokine production [50]. At this stage, transwell inserts were transferred to fresh media every 24 h.

### Pro-inflammatory Treatment

All treatments were prepared in low-serum M199-C media and added to the bottom chamber, feeding the astrocyte-lined basal side of the transwell system, while untreated low-serum M199-C media was added to the BMEC-lined apical side or upper chamber. Media changes were performed every 24 h, either with control or freshly treated low serum media. To induce A1-like polarization of astrocytes, activated microglia signaling was simulated with a pro-inflammatory cocktail. This specific formulation had been used to induce an A1-like state in rodent-derived astrocytes in vitro [9]. The treatment consisted of IL-1α (3 ng/ml; Sigma-Aldrich, St. Louis, MO, USA), TNF-α (30 ng/ml; Sigma-Aldrich, St. Louis, MO, USA), and C1q (400 ng/ml; Sigma-Aldrich, St. Louis, MO, USA). For assessment of A2-like polarization, there is currently no method for inducing this tissue injury-induced phenotype in cell culture. As such, we do not have a positive control for A2-like polarization.

### HIV-1 Tat Protein Activity Validation

To validate the activity of purified HIV-1 Tat, HepG2 cells were seeded in a 12-well plate format and transfected with the pGL3-HIV1-LTR-luciferase reporter construct (Fig. S1). The HepG2 cells were then treated with 50 ng/mL of full-length HIV-1 Tat (transactivator of transcription) (101 amino acids, ImmunoDx, Woburn, MA, USA) to assess the ability of the purified Tat protein to transactivate the HIV-1 LTR and screen for lot-to-lot variation. Tat was heat-inactivated (HIA) at 95 °C for 15 min as a negative control for LTR transactivation by purified Tat. HepG2 cells were collected 12-h post-treatment and processed for a luciferase assay as described by the manufacturer (OZ Biosciences, San Diego, CA) to assess and confirm LTR transactivation by Tat (Supplementary Fig. S1). To confirm that the purified HIV-1 Tat also exerted an effect on primary human astrocytes, whole cell lysate was harvested from cells treated daily with HIV-1 Tat at 50 ng/ml or 250 ng/ml for 4 consecutive days and a western immunoblot for GFAP using an anti-GFAP antibody (1:1000) (rabbit monoclonal antibodies; Abcam, Cambridge, UK) was performed (data not shown).

### HIV-1 Viral Protein Treatment

After functional activity of HIV-1 Tat was confirmed, to examine the effects of chronic Tat exposure, the viral protein was administered at 50 ng/ml (low dose) or 250 ng/ml (moderate-high dose) [51]. Treatment re-administration or control media replacement occurred every 24 h for up to 6 days to simulate both acute and chronic exposure. Astrocyte-conditioned media was collected from the wells or the bottom chamber in the co-culture system each day prior to media replacement and stored for subsequent analyses of astrocyte secretion profiles. Astrocyte whole cell lysate was collected from the monocultures for protein analysis and RNA extraction.

### RNA Sequencing

RNA extraction and purification was completed using the RNeasy Micro procedure as described by the manufacturer (Qiagen, Germantown, MD, USA). Following the extraction protocol, RNA purity was assessed with a spectrophotometer (Nanodrop, ND-1000) to measure absorbance at 260 nm and 280 nm, quantity by Qubit fluorometry using the RNA HS Assay procedure, and quality using Agilent Bioanalyzer nano RNA chips. RNA samples with RNA integrity number (RIN) values ≥ 8.0 were selected for sequencing. Sequencing libraries were produced using the Illumina TruSeq Stranded mRNA library preparation with poly-A selection and sequenced on an Illumina NovaSeq SP chip at 2 × 100 nt paired-end reads with a target of > 30 million clusters per sample.

### RNA Sequence Analysis

Illumina sequencing data were demultiplexed with BCL Convert v2.20.0422 as previously described to generate fastq files for each sample. These were then processed using the nf-core RNAseq pipeline v3.14.0 as previously reported [52]. In brief, this process involved read-based quality control, adapter trimming, pseudomapping reads to transcripts, and quantification of transcript abundance. The final output file was a library-size normalized measure of abundance of each transcript. This output file was used for differential gene expression analysis. To determine the effect of two different Tat concentrations, stimulation, and time-point, a linear model was employed. Using python statsmodels [53] the equation `expression ~ timepoint * stimulation * Tat` across each gene; in which time point is either 4 or 6 days, stimulation is either yes or no, and Tat is either None, Tat50, or Tat250. All items were treated as categorical. This formulation also regresses the interaction terms, determining both the independent effect of each factor and the effect of their combinations.

The effect-size and p-values were collected for each of the comparisons. Benjamini–Hochberg correction was used to account for multiple-testing; the false-discovery rate was set at 1%. Effect sizes and p-values were visualized with volcano plots. Gene lists were selected for each factor of the regression for all genes with at least an effect-size of 1.0, in either direction, and false-discovery rate below 0.01. All factors with at least 20 significant genes were submitted to Enrichr API [54]; pathways of interest are discussed below. Genes of interest representing different astrocyte polarization states were informed by the literature [8, 55]*.* Full data has been attached as a bulk file “*salmon.merged.gene_counts_scaled.tsv*”.

### Near-Infrared Western Immunoblot Analysis

Total protein content of whole-cell lysates harvested from monocultured astrocytes was first determined via a bicinchoninic acid (BCA) assay (Pierce™ BCA Protein Assay; Thermo-Fisher Scientific, Waltham, MA, USA). Samples were prepared with 25% LDS Sample Buffer (Bolt; Thermo-Fisher Scientific, Waltham, MA, USA), 10% reducing agent (Bolt; Thermo-Fisher Scientific, Waltham, MA, USA), and diluted with nuclease-free water to achieve the desired protein concentration. Samples and Chameleon Duo pre-stained protein ladder (LI-COR, Lincoln, NE, USA) were then loaded onto 4–12% Bis–Tris 1.0 mm gels (Bolt; Thermo-Fisher Scientific, Waltham, MA, USA). A mini gel tank (Thermo-Fisher Scientific, Waltham, MA, USA) filled with 1X MOPS running buffer (Bolt; Thermo-Fisher Scientific, Waltham, MA, USA) connected to a PowerPac (high current; BioRad, Hercules, CA, USA) running at 100 V was used for electrophoresis. For protein transfer to a methanol-activated PVDF membrane (LI-COR, Lincoln, NE, USA), the mini gel tank was filled with 1X transfer buffer (Bolt; Thermo-Fisher Scientific, Waltham, MA, USA) and connected to the PowerPac running at 20 V for approximately 2.5 h. Membranes were then incubated with a Tris-buffered saline (TBS)-based blocking buffer (LI-COR, Lincoln, NE, USA) for 1 h, followed by washes with 1X TBS (BioRad, Hercules, CA, USA) with 0.1% 20X TBS Tween™−20 buffer (Thermo-Fisher Scientific, Waltham, MA, USA). Primary antibodies were prepared in a solution of blocking buffer with 0.2% 20X TBS Tween™−20 and left to incubate overnight at 4°C. Human β-actin (1:2000) served as loading control (mouse monoclonal antibodies; Cell Signaling, MA, USA). A rabbit monoclonal antibody specific for human C3 (1:1000) was used to identify A1-like polarization (Abcam, Cambridge, UK). The infrared-compatible secondary antibodies used were anti-mouse IgG IRDye 680RD and anti-rabbit IgG IRDye 800CW (LI-COR, Lincoln, NE, USA), which were prepared at 1:20,000 in a solution of blocking buffer with 0.2% 20X TBS Tween™−20 and 0.03% sodium dodecyl sulfate (SDS; Millipore, Billerica, MA, USA). Blots were analyzed using the Odyssey CLx imager (LI-COR, Lincoln, NE, USA) and ImageStudio (Version 5.2). The protein of interest was measured with the 800 nm channel (green) and the housekeeping protein with the 700 nm channel (red). Band intensity was measured within ImageStudio and statistics performed within Microsoft Excel.

#### ELISA

To determine changes in astrocyte signal secretion, conditioned media samples from both monoculture experiments and from the bottom chamber within co-culture experiments were analyzed using sandwich ELISAs. ELISAs were performed for CXCL10, C3, and lipocalin2 as markers of A1-like polarization (96-well sandwich ELISA; Abcam, Cambridge, UK). ELISAs specific for S100A10 were performed to detect if A2-like polarization occurred (96-well sandwich ELISA procedure; LS-BIO, Shirley, MA, USA). Absorbance was measured using the Azure Biosystems plate reader and analyses performed with Excel.

### BBB Permeability Assay

An Evans Blue permeability assay was performed on a subset of transwells each day following treatment initiation. Evans blue-coupled albumin (EBA) was comprised of 0.5% Evans blue dye (Sigma-Aldrich, St. Louis, MO, USA), 5% BSA, and phenol red-free DMEM (Thermo-Fisher Scientific, Waltham, MA, USA). Each day, the selected transwells were first washed with phenol red-free DMEM then transferred to a new 24-well plate. The bottom chamber contained phenol red-free DMEM with 10% FBS while 0.45% EBA was added to the top chamber. The transwells were then left to incubate at 37 °C for 30 min, after which the inserts were discarded. The OD of the lower chamber was then used as a measure of barrier permeability, with higher values indicating increased passage of dye from the upper chamber to the lower chamber. As a positive control for loss of barrier integrity (approximately 0.2 OD), a small subset of transwells were treated with 1.4M of D-mannitol (Sigma-Aldrich, St. Louis, MO, USA) and allowed to incubate for 30 min prior to the Evans Blue permeability assay [46]. The contents of the basal chambers were then analyzed using a spectrophotometer (Nanodrop, ND-1000) and the amount of dye (620 nm) that passed through was quantified.

### Statistical Analysis

Unpaired Student’s t-tests were used to compare mean signal concentration levels as detected by ELISA (OD), western immunoblot band intensity (RFU), and permeability assay results (OD) for each treatment variable to the time-matched control. In assays where some results contained negative values, the data set was corrected so that the minimum value was set to 0.5, and all other values in the data set adjusted accordingly. Log_2_ fold-change calculations were performed using Excel (2016). Significance level (α) was set at 0.05.

## Results

### Primary Human Astrocytes Exposed to Simulated Activated Microglia Signaling Expressed A1-Associated Transcripts

**Fig. 1:**
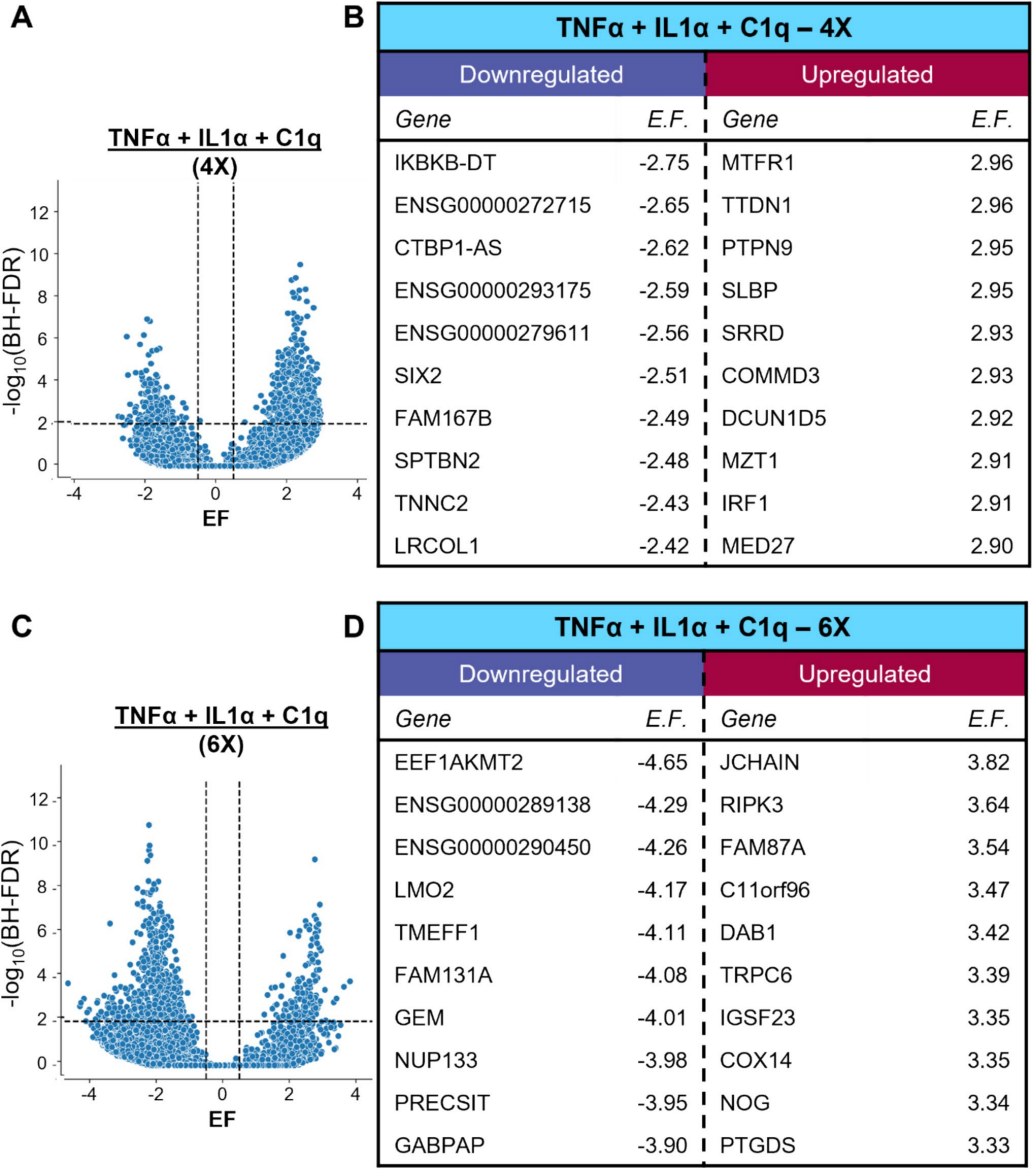
Gene expression changes in primary human fetal astrocytes following chronic exposure to TNF-α, IL-1α, and C1q (TIC). (A) Volcano plot depicting the degree of downregulation and upregulation of different genes, (B) and the top ten downregulated and upregulated transcripts in astrocytes after 4 days of treatment with TIC, (C) Volcano plot depicting degree of downregulation and upregulation of different genes, (D) and the top ten downregulated and upregulated transcripts in astrocytes after 6 days of treatment. BH-FDR = Benjamini–Hochberg False Discovery Rate; EF = Effect Size; N = 3 (per condition)

**Fig. 2:**
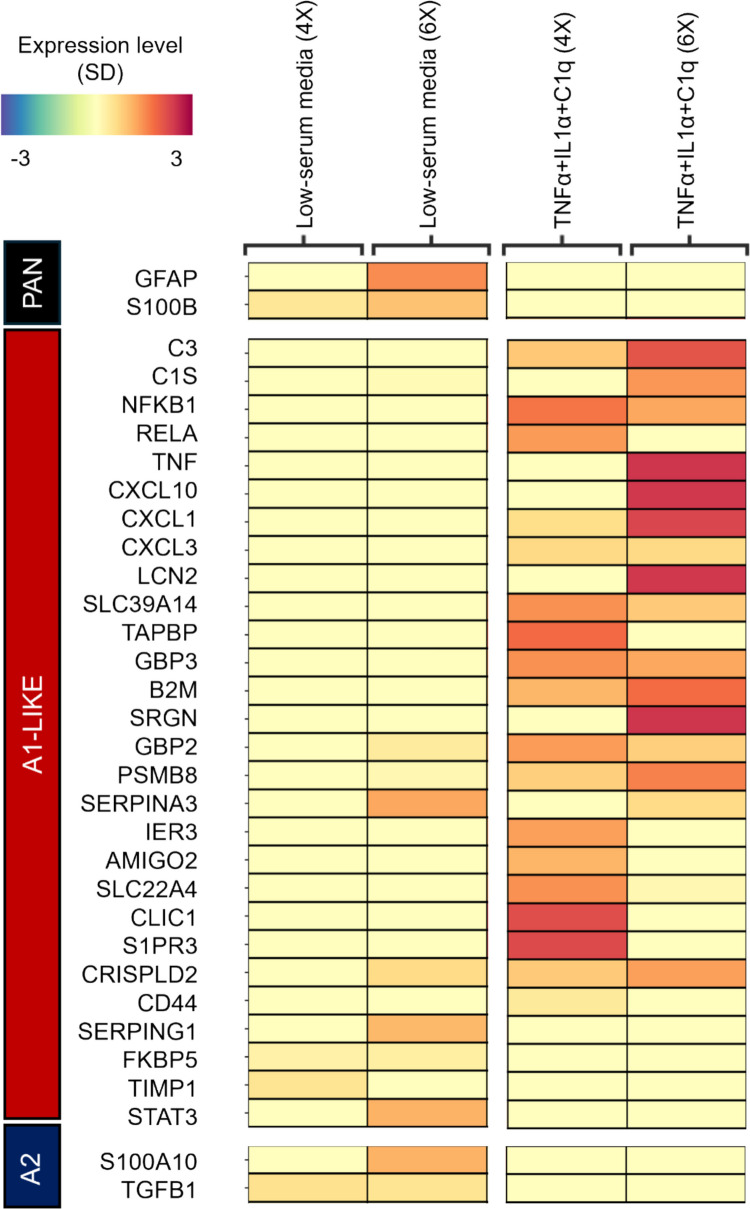
Expression of astrocyte phenotype-associated markers following chronic exposure to TIC. Heatmap depicting expression level for common markers of pan-reactive, A1-associated, and A2-associated genes. Values were z-scaled by gene with blue representing expression below average and magenta indicating above average expression. Expression levels are depicted by change in standard deviation (−3 to 3). Each column represents the average expression from 3 samples

To determine if primary human astrocytes cultured in vitro could be induced to polarize to a neuroinflammatory A1 or A1-like astrocyte phenotype as observed in animal cell models, astrocytes were exposed to a cocktail of the molecules normally secreted by activated microglia: TNF-α (30 ng/ml), IL-1α (3 ng/ml), and C1q (400 ng/ml), abbreviated as TIC, using dosages adopted from animal cell studies [9]. There are several dozen genes that have been noted to have upregulated RNA levels in A1- or A1-like polarized astrocytes; indeed, it was this coordinated gene upregulation that first identified this phenotype. Primary human astrocytes that underwent repeated treatment with TIC every day for 4- or 6-days were harvested and processed to obtain purified RNA. Compared to control, after daily treatments for 4 days, primary human astrocytes displayed significant changes in the expression of 762 genes (Fig. 1A, B). After daily treatments for 6 days (approaching the maximum 9-day limit for primary human astrocyte culture), there were significant changes in the expression of 682 transcripts (Fig. 1C, D). According to the Human ncRNA Database (GeneCaRNA), several long noncoding RNA (lncRNA) and novel transcripts with currently undefined or uncharacterized functions were found to be significantly downregulated following chronic exposure to pro-inflammatory stimulation [56, 57] (Fig. 1B, D). Gene ontological analyses of the significantly differentially expressed genes showed enrichment of pathways associated with immune responses at 4 days of treatment, while 6 days of treatment promoted pathways associated with cellular modification of the extracellular space (Table 1). This was consistent with changes seen in control cells after 4 days compared to 6 days, suggesting that primary human astrocytes tended to upregulate pathways associated with extracellular interactions and structural modifications over time, irrespective of stimulatory treatments (Supplementary Fig. S2A–B). A heatmap comparing gene expression profiles was generated to determine if chronic exposure to TIC induced a change in phenotype according to the A1/A2 paradigm, looking at commonly-used transcriptional markers of A1- and A2-like astrocytes [8] (Fig. 2, Supplementary Table 1). Pan-reactive markers, including GFAP, were not increased in response to pro-inflammatory signaling. There were also no significant changes in A2-associated markers S100A10 and TGFB1 (Fig. 2). A1-associated markers, including C3, NFKB1, CXCL10, CXCL1, CXCL3, LCN2, and SLC39A14, were significantly increased following chronic exposure to TIC after both 4 and 6 days of treatment (Fig. 2). Many of these genes encode proteins associated with pro-inflammatory responses. These results confirmed that simulated microglia signaling treatment successfully induced A1-like polarization in cultured primary human fetal cells.

**Table 1:** Gene enrichment pathway analysis. *N* = 3 (per condition)

Enriched pathway	*Adjusted P*
TNFα + IL1α + C1q (4X)	
Regulation Of Cell Communication	0.0003
Positive Regulation of Nucleocytoplasmic Transport	0.0091
Cellular Response to Lipopolysaccharide	0.0030
Cellular Response to Molecule of Bacterial Origin	0.0037
Response to Lipopolysaccharide	0.0037
Regulation of I-κB kinase/NF-κB Signaling	0.0037
Inflammatory Response	0.0040
Positive Regulation of Cell Migration	0.0031
Negative Regulation of Cell Population Proliferation	0.0003
TNFα + IL1α + C1q (6X)	
Extracellular Structure Organization	0.0002
External Encapsulating Structure Organization	0.0002
Extracellular Matrix Organization	0.0006
Transmembrane Receptor Tyrosine Kinase Signaling Pathway	0.0034
Positive Regulation of MAPK Cascade	0.0045

### Chronic Exposure of Primary Human Astrocytes to Simulated Activated Microglia Signaling Induced Polarization to an A1-Like Phenotype

**Fig. 3:**
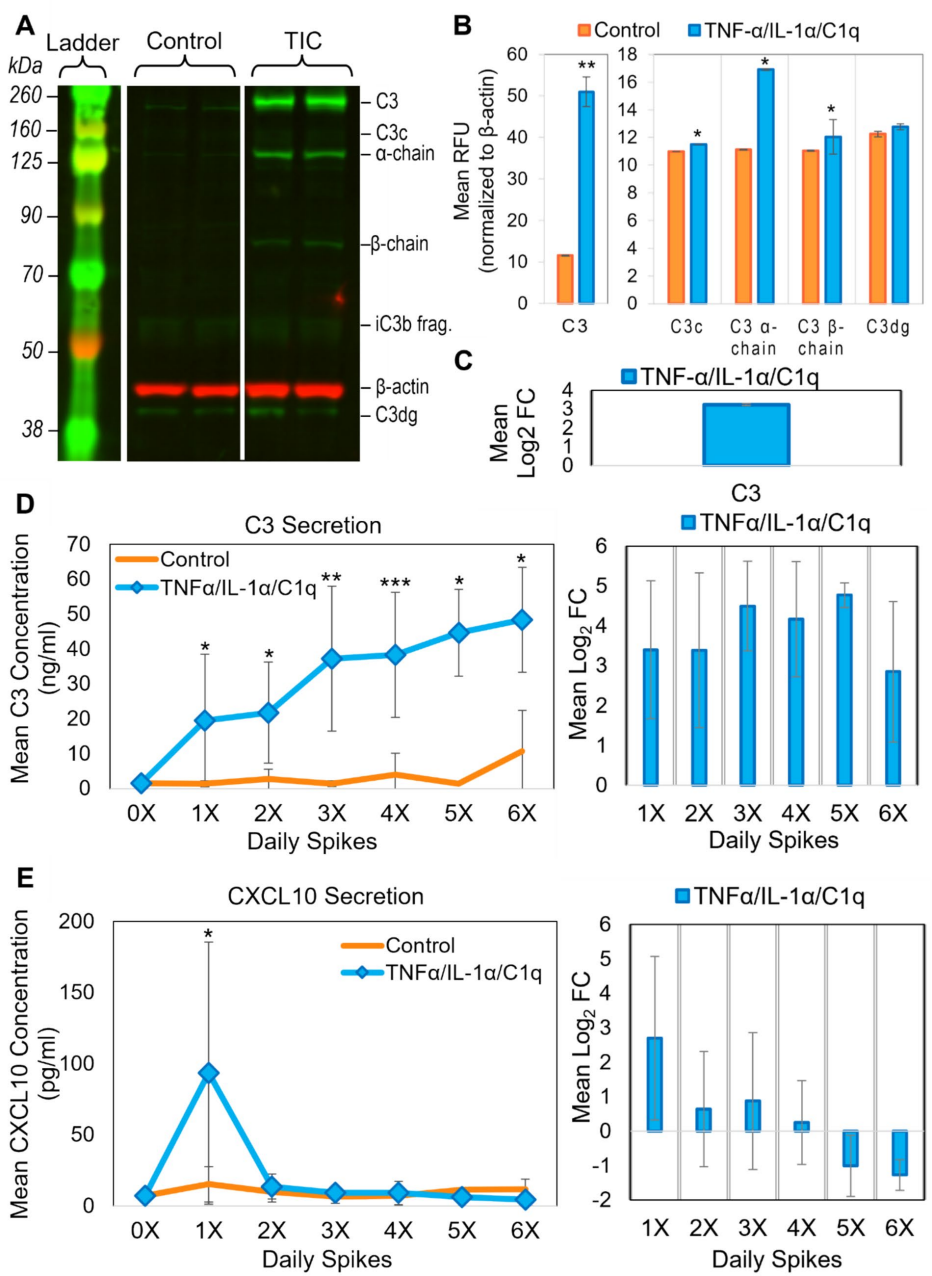
Chronic exposure of primary human astrocytes to TIC induced increased C3 protein expression and secretion of pro-inflammatory signals indicative of an A1-like phenotype. (A) Representative near-infrared (IR) western immunoblot on whole cell lysate following 4 days of treatment with TIC showed increased expression of A1-associated C3 (green) and its cleavage products. Total protein (5 µg) loaded per lane, with β-actin (red) serving as a loading control. (B) Quantification of mean relative fluorescence intensity of C3 in representative western blots, with band intensity values corrected and normalized to β-actin. (C) Mean log_2_ fold change of intact C3 expression following TIC treatment compared to control from several blots (n = 3 biological replicates). (D) ELISA measuring C3 concentration (ng/ml) and mean log_2_ fold change in astrocyte-conditioned media harvested each day prior to daily TIC spike and at the conclusion of the experimental time course. The 0X timepoint is represented by an experimental n = 4 and technical n = 7. (E) ELISA measuring CXCL10 concentration (pg/ml) and mean log_2_ fold change. The 0X timepoint is represented by an experimental n = 3 and technical n = 5. OD measured in duplicate at 450 nm and corrected for negative values within each set of assay results. Plotted is the mean of experimental replicate averages. In panels D and E, control values are represented by orange lines (1X: experimental n = 7, technical replicate n = 12; 2X: experimental n = 7, technical replicate n = 13; 3X: experimental n = 7, technical replicate n = 12; 4X: experimental n = 7, technical replicate n = 13; 5X: experimental n = 2, technical replicate n = 4; 6X: experimental n = 3, technical replicate n = 6). TIC-treated sample values represented by blue diamonds (1X: experimental n = 7, technical replicate n = 12; 2X: experimental n = 7, technical replicate n = 13; 3X: experimental n = 8, technical replicate n = 14; 4X: experimental n = 7, technical replicate n = 13; 5X: experimental n = 3, technical replicate n = 6; 6X: experimental n = 3, technical replicate n = 6). For all assays, statistical significance was determined using Student’s t-test comparing untreated to treated corrected mean values. Error bars represent SD. *p ≤ 0.05, **p ≤ 0.005, ***p ≤ 0.0005

The neuropathological effects of A1 or A1-like astrocytes are most relevant in chronic neurological diseases, as opposed to acute conditions like ischemic injury. While a noticeable increase in the A1-associated marker C3 was elicited after only 24 h of treatment, this effect becomes greater with repeated treatments (Supplementary Fig. S3). As such, the experimental protocol involved replacing cell media, in the absence or presence of freshly prepared treatment, approximately every 24 h, with treatment courses ranging from 3 to 6 days. To confirm that the changes observed in RNA expression profiles correlated with changes in protein expression, near-infrared western immunoblot analysis was performed on whole cell lysate collected from untreated and treated astrocytes. Given its consistent use in the reactive astrocyte literature, C3 was used as the primary protein marker. Intact C3 (~ 195 kDa) was often cleaved into different functional isoforms, including the α-chain (~ 120 kDa) and β-chain (~ 75 kDa), which were subsequently cleaved into smaller fragments [58]. Astrocytes treated with TIC, for 4 days displayed a significant increase in intracellular expression of intact C3, as well as several C3 isoforms (Fig. 3A–C). As the C3 isoforms like C3c, C3dg, and iC3b fragments were cleaved from intact C3 during production of functional complement, this suggested that the cells not only transcribed C3, but had also prepared to secrete it [58]. As A1-like astrocytes help establish a pro-inflammatory environment, the signal secretion profiles of A1-like primary human fetal astrocytes were characterized over the course of exposure to simulate microglia signaling. Using astrocyte-conditioned media collected each day, ELISAs were performed to quantify the concentration of C3 and CXCL10 (Fig. 3D, E). Consistent with intracellular protein expression changes, the complement component C3 was secreted by astrocytes in response to simulated microglia signaling, with the concentration secreted each day rising over time (Fig. 3D). Secretion of CXCL10, a chemoattractant for immune cells, was significantly upregulated within the first 24 to 48 h of exposure to TIC compared to control, and then appeared to level off (Fig. 3E). Secretion of an additional A1-associated marker, lipocalin2, was also significantly increased in treated cells (Supplementary Fig. S4A). In contrast, the A2-associated marker S100A10 was not detected (Supplementary Fig. S5A). These results, in conjunction with the observed changes in gene expression (Supplementary Fig. S6), indicated that daily exposure to TIC induced an A1 or A1-like polarization in primary human fetal astrocytes and resulted in increased secretion of pro-inflammatory factors.

### Astrocytes Exposed to Both Low and High Doses of HIV-1 Tat Did Not Significantly Express A1-Associated Markers at the RNA Level

**Fig. 4:**
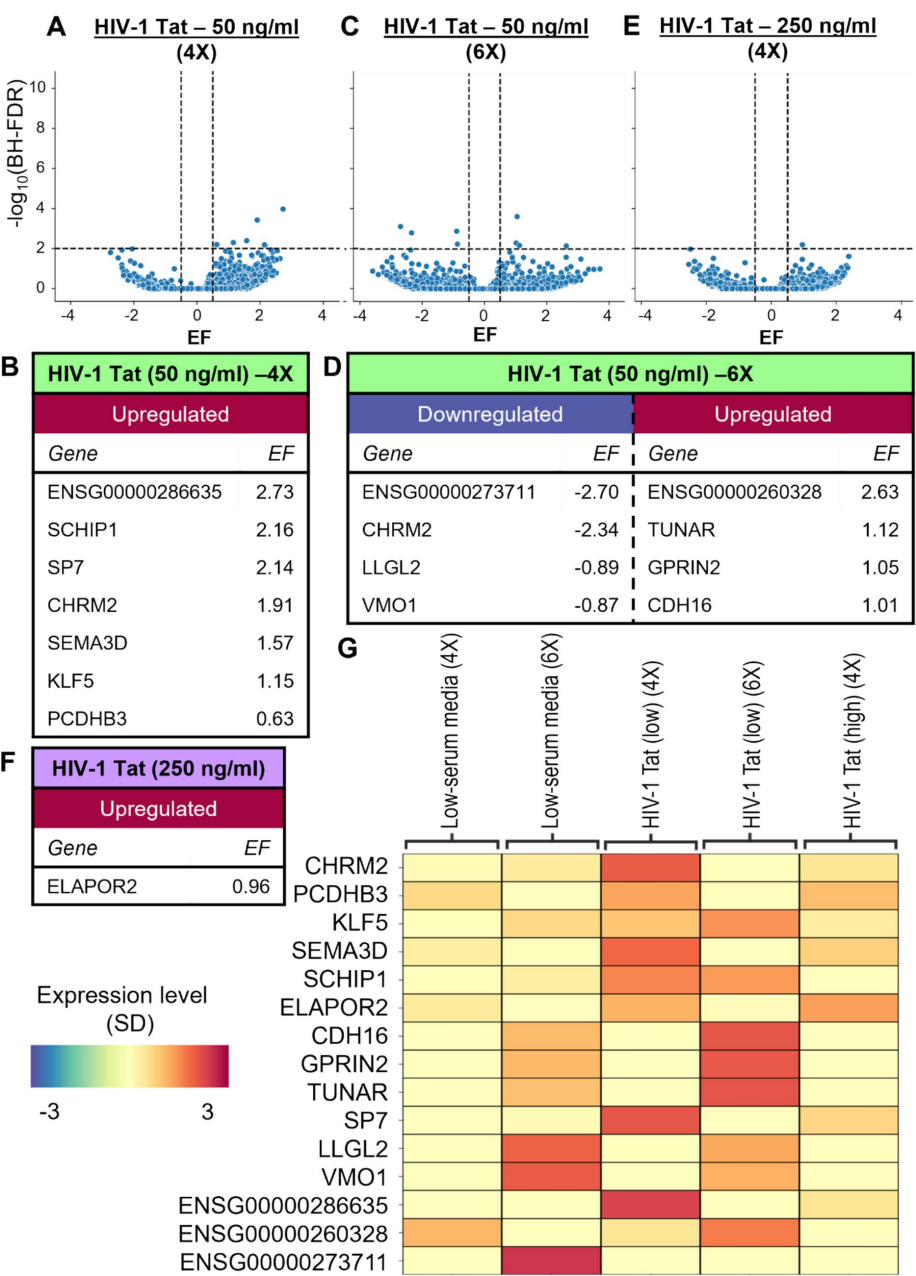
Gene expression changes in primary human fetal astrocytes following chronic exposure to HIV-1 Tat. **(A)** Volcano plot depicting degree of downregulation and upregulation of different genes **(B)** and transcripts with the most significant expression changes in astrocytes after 4 days of treatment with HIV-1 Tat (50 ng/ml). **(C)** Volcano plot depicting the regulation of different genes **(D)** and transcripts with the most significant expression changes in astrocytes after 6 days of treatment with HIV-1 Tat (50 ng/ml). **(E)** Volcano plot depicting gene regulation **(F)** and transcripts with the most significant upregulation in astrocytes after 4 days of treatment with a high dosage of HIV-1 Tat (250 ng/ml). **(G)** Heatmap comparing HIV-1 Tat-induced expression changes across different dosages and timepoints. Values were z-scaled by gene with blue representing expression below average and magenta indicating above average expression. Expression levels are depicted by change in standard deviation (−3 to 3). Each column represents the average expression from 3 samples. BH-FDR = Benjamini–Hochberg False Discovery Rate; EF = Effect Size

Several neuropathological proteins have been observed to induce A1 or A1-like polarization independent of microglia signals, including in Alzheimer’s disease and *Toxoplasma gondii* infection [2, 13, 15]. The role of A1-like astrocytes has not been well characterized in infectious diseases that target the nervous system, with viral diseases being particularly under-studied. HIV-1 is a chronic disease that can contribute to neurodegenerative disorders, such as HAND. Even during latent infection, viral proteins like Tat are still constitutively expressed by infected cells in the CNS, and these pathogenic proteins are known to activate microglia, which go on to secrete pro-inflammatory factors that might activate astrocytes [39–41]. To determine if HIV-1 Tat can directly activate astrocytes and induce an A1-like polarization independent of microglia, astrocytes were treated with two different dosages of purified free HIV-1 Tat: 50 ng/ml as a low dose and 250 ng/ml as a high dose. To achieve a more comprehensive picture of the changes undergone by astrocytes following exposure to HIV-1 Tat, RNA-seq analysis was performed on samples collected after daily treatments for 4 days, and for the low dosage of Tat, at 6 days of treatments (Fig. 4). Astrocytes chronically exposed to HIV-1 Tat only displayed significant upregulation in the expression of a select number of genes. Following 4 days of treatment with HIV-1 Tat at a low dose, astrocytes displayed significant upregulation of 7 genes (Fig. 4A, B, G). After 6 days, only 4 genes were upregulated, and 4 genes were downregulated (Fig. 4C, D, G). According to GeneCaRNA, 3 of these genes were lncRNAs that are currently poorly characterized [56, 57]. Treatment with a higher dose of HIV-1 Tat only significantly influenced the expression of 1 gene, ELAPOR2, which is thought to be a regulator of the bone morphogenetic protein (BMP) signaling pathway [59] (Fig. 4E–G). Of these significantly altered genes, none were considered markers of A1-like astrocyte activation (Supplementary Fig. S7 and S8).

### Chronic Exposure to HIV-1 Tat Did Not Independently Induce A1-Like Polarization

**Fig. 5:**
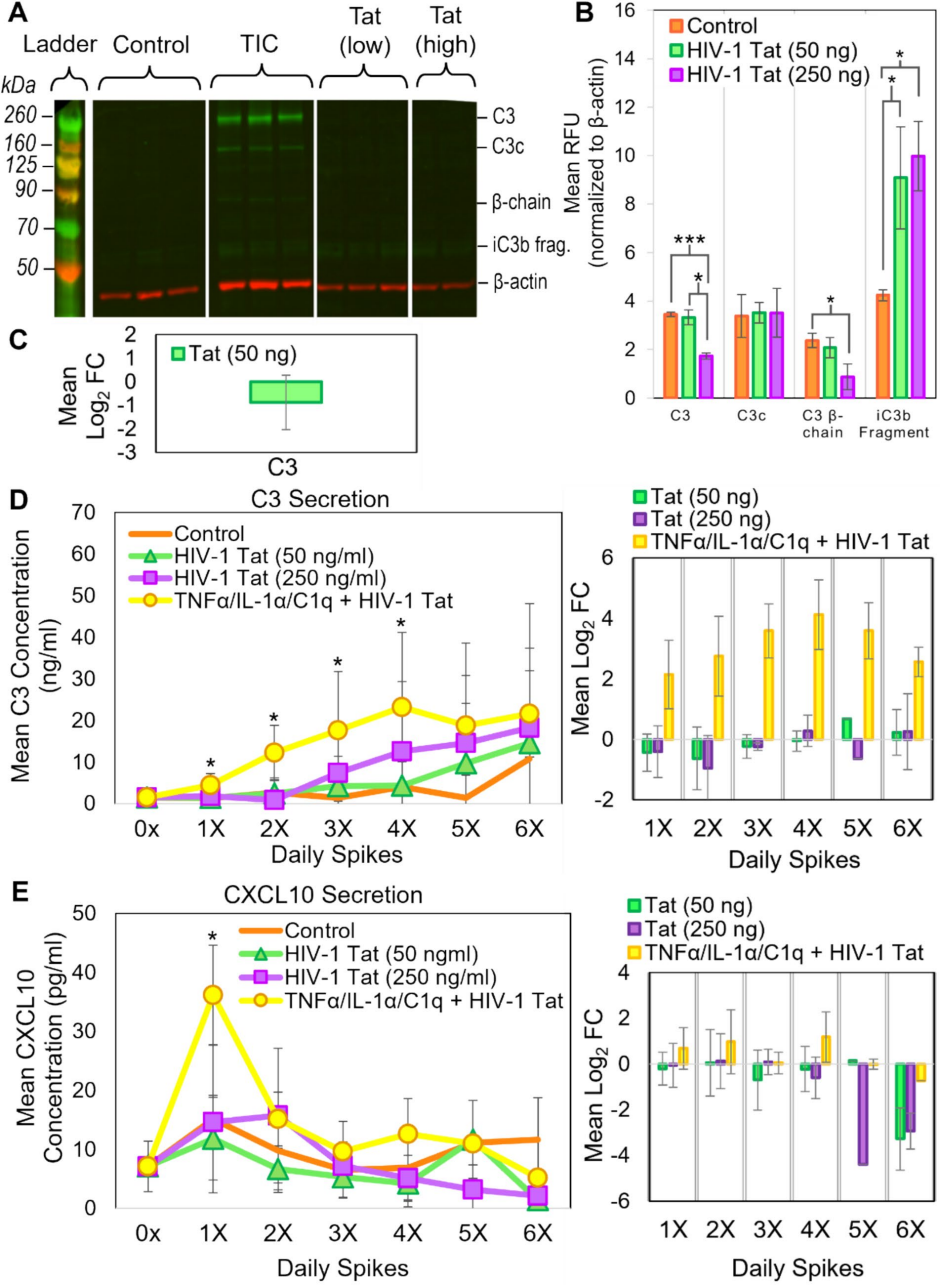
Chronic exposure of primary human astrocytes to HIV-1 Tat did not independently induce significantly increased expression of A1-associated markers or pro-inflammatory signal secretion. **(A)** Representative near-infrared (IR) western blot on whole cell lysate following 4 days of treatment with HIV-1 Tat at 50 ng/ml and 250 ng/ml, with TIC treatment displayed as a positive control. No significant increase in intact C3 (green), and only some C3 cleave products detected. 5 µg total protein loaded per lane, with β-actin (red) serving as a loading control. **(B)** Quantification of mean relative fluorescence intensity of C3 in representative western blot, with band intensity values corrected and normalized to β-actin. **(C)** Mean log_2_ fold change of intact C3 expression following HIV-1 Tat (50 ng/ml) exposure compared to control from several blots (n = 2 biological replicates). **(D)** ELISA measuring C3 concentration (ng/ml) and mean log_2_ fold change in astrocyte-conditioned media harvested each day prior to daily spike and at the conclusion of the experimental time course. The 0X timepoint is represented by an experimental n = 4 and technical n = 7. **(E)** ELISA measuring CXCL10 concentration (pg/ml) and mean log_2_ fold change. The 0X timepoint is represented by an experimental n = 3 and technical n = 5. OD measured in duplicate at 450 nm and corrected for negative values within each set of assay results. Plotted is the mean of experimental replicate averages. In panels D and E, control values are represented by orange lines (see Fig. 3). HIV-1 Tat (50 ng/ml) was represented by green triangles (1X: experimental n = 5, technical replicate n = 10; 2X: experimental n = 6, technical replicate n = 12; 3X: experimental n = 6, technical replicate n = 12; 4X: experimental n = 6, technical replicate n = 12; 5X: experimental n = 2, technical replicate n = 4; 6X: experimental n = 2, technical replicate n = 4). HIV-1 Tat (250 ng/ml) was represented by purple squares (1X: experimental n = 4, technical replicate n = 7; 2X: experimental n = 4, technical replicate n = 8; 3X: experimental n = 4, technical replicate n = 7; 4X: experimental n = 3, technical replicate n = 5; 5X: experimental n = 2, technical replicate n = 4; 6X: experimental n = 2, technical replicate n = 4). Samples treated with TIC + HIV-1 Tat (50 ng/ml) are represented by yellow circles. (1X: experimental n = 3, technical replicate n = 6; 2X: experimental n = 4, technical replicate n = 8; 3X: experimental n = 4, technical replicate n = 8; 4X: experimental n = 4, technical replicate n = 8; 5X: experimental n = 2, technical replicate n = 4; 6X: experimental n = 2, technical replicate n = 4). For all assays, statistical significance determined using Student’s t-test comparing untreated to treated corrected mean values. Error bars represent SD. *p ≤ 0.05, **p ≤ 0.005, ***p ≤ 0.0005

To determine if chronic exposure to HIV-1 Tat could stimulate production of A1-associated signals independent of simulated microglia signaling, astrocytes were treated with low and high doses of HIV-1 Tat, as well as a combination of TIC and HIV-1 Tat (50 ng/ml) to serve as a more representative model of a chronic low-level inflammatory environment in the context of HIV-1 infection. Whole cell lysates were analyzed by near-infrared western blot (Fig. 5A–C). The A1 phenotype-associated marker C3 was found to not be significantly increased following treatment with HIV-1 Tat alone; instead, the level of stable C3 was markedly lower in cells treated with high doses of HIV-1 Tat compared to control (Fig. 5C). The only variant of C3 that was found to be increased were fragments of iC3b [60]. Astrocyte-conditioned media from the different treatment protocols was collected and analyzed via ELISA in order to characterize changes in astrocyte secretion profiles. No significant change in secretion of C3 or CXCL10 was observed following exposure to either dosage of HIV-1 Tat (Fig. 5D, E). When cells were treated with TIC combined with a low dose of Tat, there was a significant increase in C3 and CXCL10 secretion; therefore, HIV-1 Tat exposure did not extinguish the activating effect of simulated microglia signaling (Fig. 5D, E). Secreted lipocalin2 was not increased in response to chronic exposure to HIV-1 Tat at low doses alone (Supplementary Fig. S4B). To confirm that HIV-1 Tat exposure did not induce an A2-like phenotype, an ELISA for the A2-associated marker S100A10 was performed, resulting in no detectable secretion (Supplementary Fig. S5B). Similarly, at the RNA level, other A2-associated markers such as TGFB1 and BDNF were not found to be increased HIV-1 Tat-treated cells after 4 days of treatment (Supplementary Fig. S7 and S8). In conclusion, chronic exposure to varying doses of HIV-1 Tat failed to elicit a significant pro-inflammatory response in primary human astrocytes, in contrast to treatment with TIC (Figs. 1, 2, 3, Supplementary Fig. S6). This suggests that free HIV-1 Tat alone was unable to induce A1-like polarization independently of microglia signaling in this particular experimental system.

### Astrocytes Co-cultured with BMECs in an In Vitro Human BBB Model Significantly Upregulated Secretion of A1-Associated Inflammatory Signals Following Chronic Exposure to Simulated Activated Microglia Signaling

**Fig. 6:**
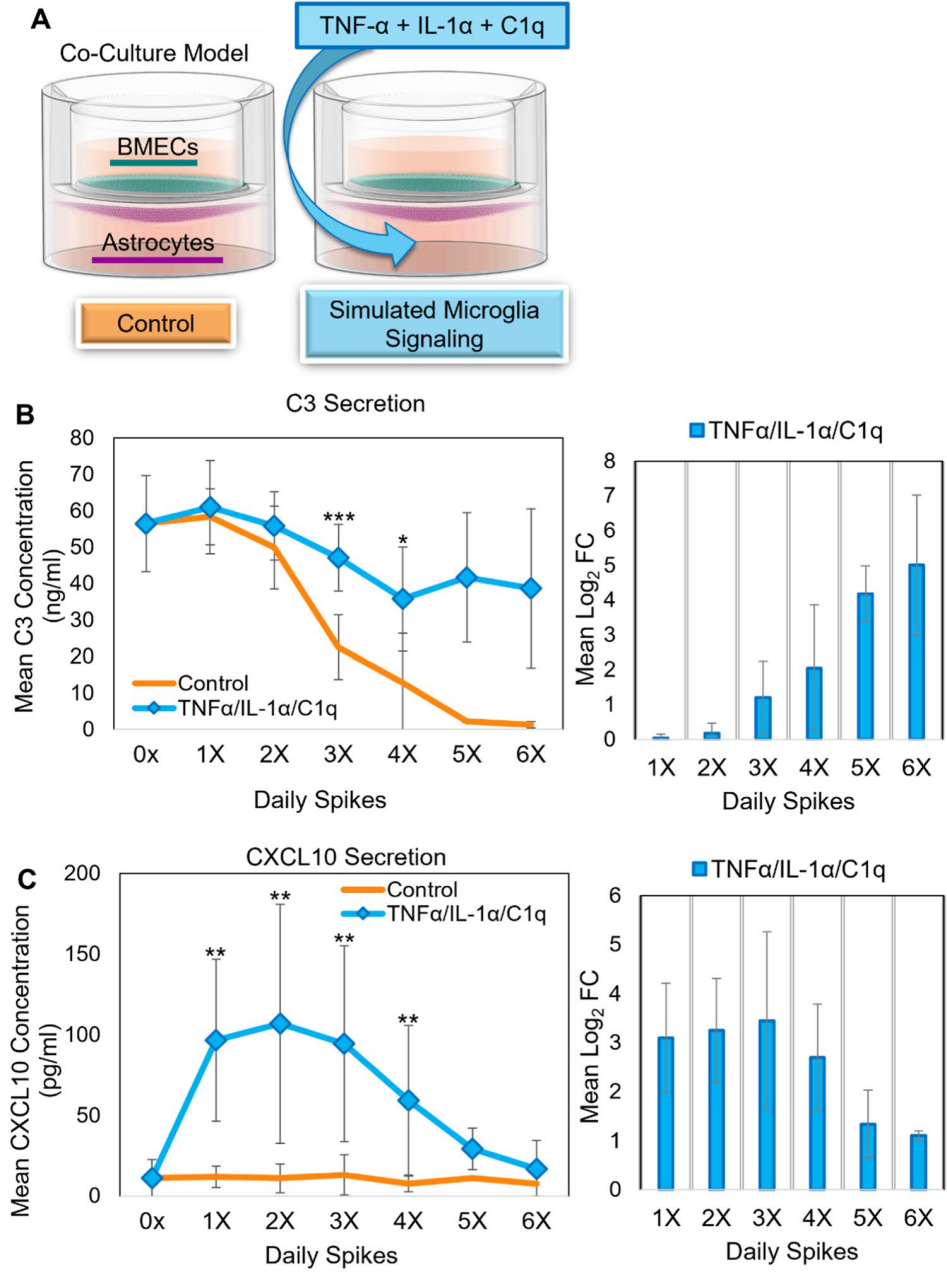
Primary human astrocytes co-cultured with primary human BMECS in an in vitro BBB model upregulated A1-associated pro-inflammatory signals following chronic treatment with TIC. **(A)** Diagram of transwell co-culture system. Transwell inserts are seeded with astrocytes on the basal side and BMECs on the apical side (day 0). Treatment with low-serum media (control) or TIC begins on day 3 (0X) and occurs every 24 h until day 9 (6X). Conditioned media from basal chambers were harvested each day prior to daily spike. ELISA analysis was performed to determine expression profiles of A1 astrocyte-associated pro-inflammatory signals. **(B)** ELISA measuring C3 concentration (ng/ml) and mean log_2_ fold change in astrocyte-conditioned media harvested from BBB model each day prior to daily spike and at the conclusion of the experimental time course. **(C)** ELISA measuring CXCL10 concentration (pg/ml) and mean log_2_ fold change in astrocyte-conditioned media in the co-culture model. OD measured in duplicate at 450 nm and corrected for negative values within each set of assay results. Plotted is the mean of experimental replicate averages. In panels D and E, the 0X timepoint is represented by an experimental n = 5 and technical n = 16. Control values are represented by orange lines (1X: experimental n = 6, technical replicate n = 14; 2X: experimental n = 6, technical replicate n = 14; 3X: experimental n = 7, technical replicate n = 14; 4X: experimental n = 7, technical replicate n = 11; 5X: experimental n = 3, technical replicate n = 8; 6X: experimental n = 3, technical replicate n = 7). TIC-treated sample values represented by blue diamonds (1X: experimental n = 5, technical replicate n = 13; 2X: experimental n = 6, technical replicate n = 14; 3X: experimental n = 7, technical replicate n = 14; 4X: experimental n = 7, technical replicate n = 12; 5X: experimental n = 3, technical replicate n = 8; 6X: experimental n = 3, technical replicate n = 7). Statistical significance determined using Student’s t-test comparing untreated to treated corrected mean values. Error bars represent SD. *p ≤ 0.05, **p ≤ 0.005, ***p ≤ 0.0005

The BBB is comprised of tightly packed BMECs that ensheath cerebral blood vessels. Astrocytes are a crucial component in maintaining the functional integrity of this barrier. To investigate if A1-like astrocyte polarization could occur in barrier-associated astrocytes, a transwell model of the BBB comprised of primary human BMECs and astrocytes seeded on opposite sides of a permeable membrane was used (Fig. 6A). In this in vitro model of the BBB, the upper BMEC-lined chamber represented the blood vessels, and the lower astrocyte-lined chamber represented the brain parenchyma. To determine if exposure to simulated microglia signaling would induce A1-like polarization in barrier-associated astrocytes co-cultured with BMECs, the astrocyte-lined bottom chamber of selected transwells was treated with TIC every 24 h for up to 6 days. Using astrocyte-conditioned media collected from the bottom chambers of the in vitro BBB models, ELISAs for A1-associated markers were performed. In the untreated control, secretion of C3 was detected at high levels at day 0, but lowered to undetectable levels by day 5 of treatment. In contrast, the TIC-treated transwells maintained a high level of secretion throughout the treatment course (Fig. 6B). CXCL10 secretion remained low in the control but spiked following the initial TIC treatment, and gradually returned to baseline after several days (Fig. 6C). Lipocalin2 secretion was also significantly increased after chronic exposure to simulated microglia signaling (Supplementary Fig. S4C). The A2 astrocyte marker S100A10 was not detected in the co-culture model, indicating that the cells did not adopt an A2-like phenotype (Supplementary Fig. S5C). These findings suggested that exposure to simulated microglia signaling induced a C3 + A1-like polarization response in primary human astrocytes co-cultured with BMECs in an in vitro BBB model.

### Chronic Exposure to the Viral Protein HIV-1 Tat Did Not Significantly Upregulate Secretion of A1-Associated Pro-inflammatory Signals in Astrocytes Co-cultured with BMECs in an In Vitro BBB Model

Next, the co-culture system was exposed to both low and high chronic doses of HIV-1 Tat to determine if the viral protein could independently induce an A1-like response in barrier-associated astrocytes (Supplementary Fig. S9A). Astrocyte-conditioned media was harvested every 24 h and analyzed via ELISA. Secretion of C3 was detected at high levels from the beginning of the experiment, but returned to baseline after several days, despite exposure to HIV-1 Tat (Supplementary Fig. S9B). CXCL10 concentration did not significantly increase at any point following HIV-1 viral protein exposure (Supplementary Fig. S9C). Lipocalin2 secretion did not significantly increase with HIV-1 Tat exposure (Supplementary Fig. S4D). The A2 marker S100A10 was not detected (Supplementary Fig. S5D). These findings suggest that HIV-1 Tat does not induce A1-like polarization responses in astrocytes associated with a BBB on its own, but instead stimulates astrocytes through activation of microglia.

###  Permeability of an In Vitro BBB Model Was Not Significantly Impaired by Chronic Exposure to Simulated Microglia Signaling nor Low or High Doses of HIV-1 Tat 

**Fig. 7:**
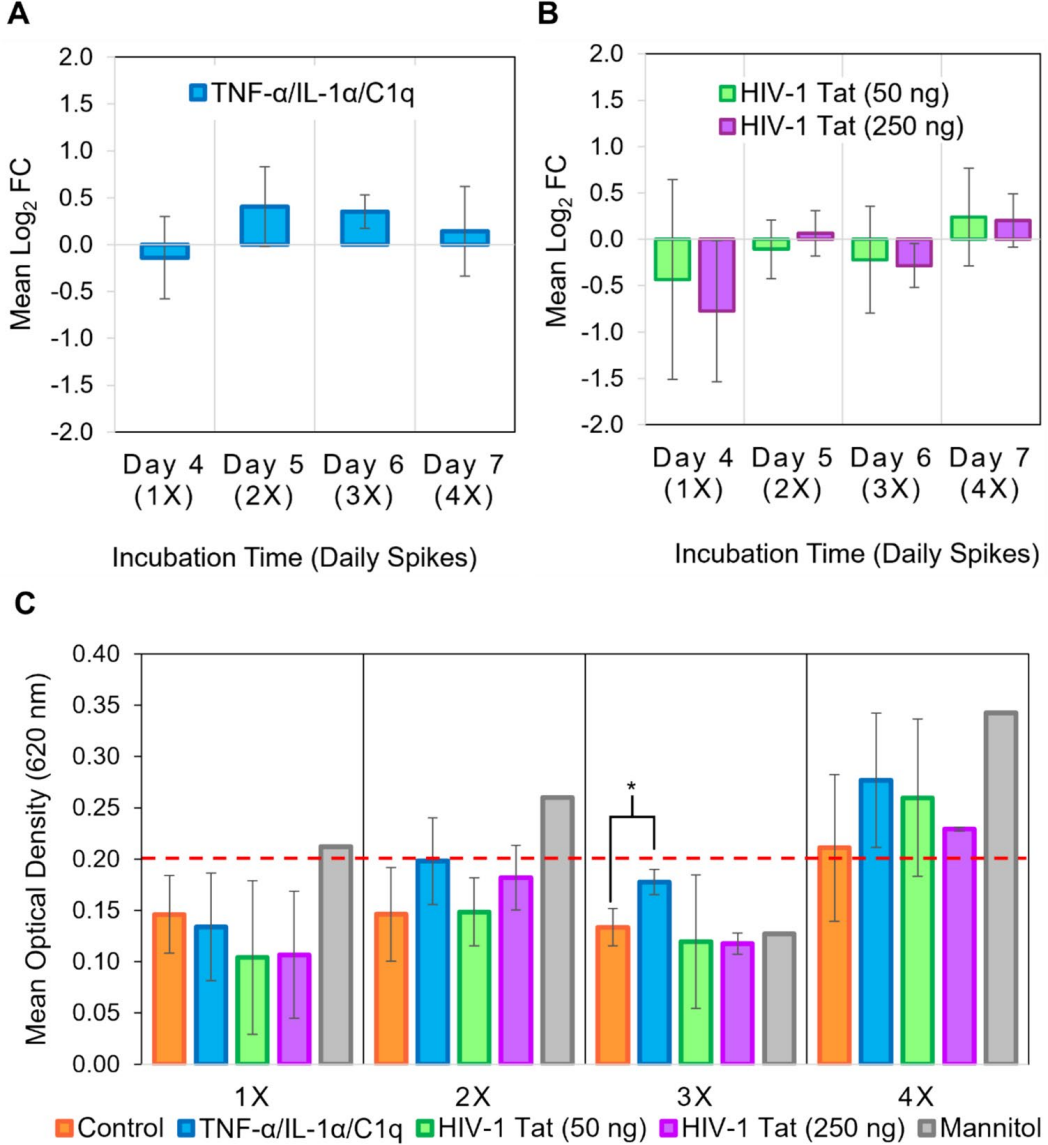
No significant changes in permeability of in vitro BBB model following chronic exposure to simulated activated microglia signaling or HIV-1 Tat. Transwell inserts were seeded with primary human fetal astrocytes on the basal side and primary human BMECs on the apical side at day 0. Treatment with low-serum media (control), TIC, HIV-1 Tat (50 ng/ml), or HIV-1 Tat (250 ng/ml) began on day 3 and continued for 4 days. Transwells (n = 1–3 per experiment) were selected each day to undergo an Evans Blue permeability assay. Inserts were washed with PBS and Evans Blue-coupled albumin prepared in phenol red-free DMEM was added to the apical chamber and left to incubate for 30 min, after which the optical density (OD) of the basal chamber was analyzed by spectrometry at 620nm. (**A)** Mean log_2_ fold change of dye permitted to pass through barrier in co-culture models treated with simulated microglia signaling compared to control. Experimental n = 4–6 and total technical replicate n = 8–18 **(B)** Mean log_2_ fold change of dye permitted to pass through barrier in transwells treated with low dose HIV-1 Tat (50 ng/ml) (green), or high dose HIV-1 Tat (250 ng/ml) (purple) compared to control. Experimental n = 2–4 and total technical replicate n = 4–8. **(C)** Mean absorbance across 2–4 trials. Mannitol (gray) represents positive control for barrier breakage. Error bars represent SD. *p ≤ 0.05

To determine if exposure to simulated activated microglia signaling and A1-like astrocyte polarization would have consequences for overall permeability of the BBB, an Evans Blue permeability assay was performed every 24 h following treatment, with mannitol-exposed subset of transwells as a positive control [46]. Over the course of 4 days of repeated TIC treatments, a significant change in barrier permeability was not observed (Fig. 7A, C), suggesting that exposure to TIC did not ultimately impair astrocyte-associated barrier structures compared to untreated barriers. To determine if chronic exposure of barrier-associated astrocytes to low or high doses of HIV-1 Tat would influence the structural integrity of the barrier, an Evans Blue permeability assay at 24-h intervals was performed. No significant increase in dye passage across the transwell barrier was observed (Fig. 7B, C), suggesting that chronic exposure to HIV-1 Tat also did not directly impair BBB integrity.

## Discussion

**Fig. 8:**
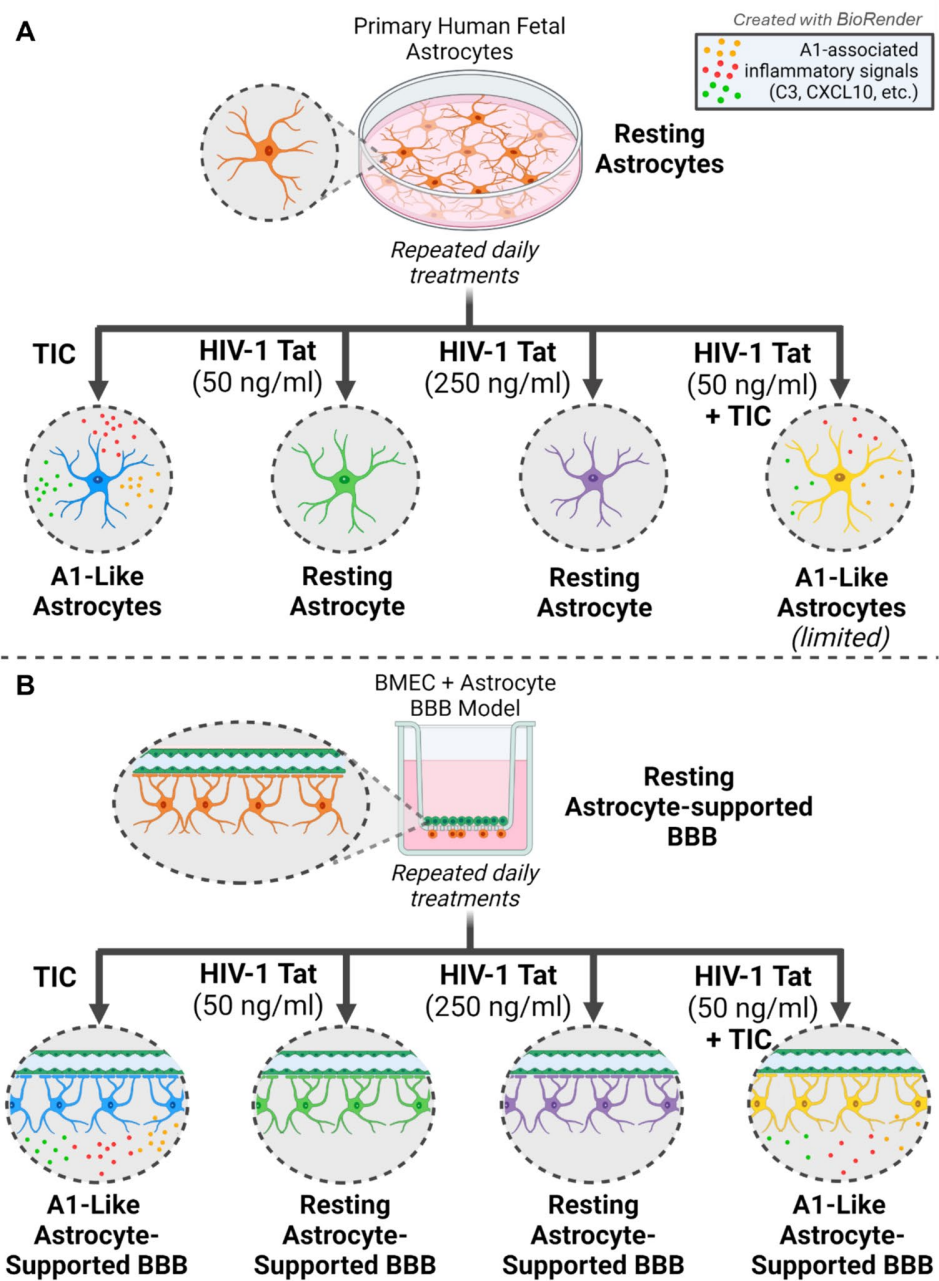
Simulated microglia signaling, but not HIV-1 Tat, induces an A1-like phenotype in primary human astrocytes in monoculture and in a co-culture BBB model. **(A)** Chronic exposure to TIC treatment (TNFα, IL-1α, and C1q) simulating activated microglia signaling promoted primary human astrocytes to polarize to an A1-like phenotype that secreted pro-inflammatory factors, including C3 and CXCL10. Treatment with different doses of purified free HIV-1 Tat does not independently induce this reaction, while a combination of HIV-1 Tat and TIC appears to induce a less robust A1-like polarization response. **(B)** TIC treatment of astrocytes in a primary human fetal cell model of the BBB, comprised of astrocytes and BMECs seeded on opposite sides of a transwell membrane, induces barrier astrocytes to secrete A1-associated markers, indicated pro-inflammatory phenotype polarization. HIV-1 Tat exposure without TIC did not elicit A1-like polarization of barrier astrocytes

Neurotoxic reactive astrocytes and their role in various neuropathologies represent a growing area of active research. To better understand A1-like phenotypes in the context of various human diseases, we developed and validated an in vitro model using primary human fetal astrocytes. Primary human cells are more physiologically representative than immortalized cell lines; however, variations in fetal gene expression compared to mature cells can present challenges. To determine if cultured fetal cells are capable of displaying a distinct polarization response, as has been observed in rodent cells and adult human tissue samples, astrocytes were treated daily with a cocktail of the signals secreted by activated microglia: TNF-α, IL-1α, and C1q (TIC) (Fig. 8). RNA sequencing confirmed that many of the genes upregulated in A1-like astrocytes were also upregulated in the primary human fetal astrocyte model, suggesting polarization to an A1-like phenotype [8] (Fig. 2). When comparing gene expression profiles of human fetal cells to validated rodent cells as described in [8], transcriptional variation between species is to be expected among reactive astrocytes. Indeed, several genes that are highly upregulated in rodent A1 astrocytes were not upregulated in primary human fetal astrocyte, including TIMP1 (tissue inhibitor of metalloproteinases 1), which was highly expressed in all conditions, and SERPING1 (serpin family G member 1), which was decreased in primary human fetal astrocytes treated with simulated microglia signaling. Interestingly, the protein encoded by SERPING1 is associated with regulatory inhibition of the complement system and may be upregulated in CD14 + monocytes from PLWH as a mechanism of limiting infection [61]. A trend of upregulation of SERPING1 transcripts was observed in astrocytes treated with HIV-1 Tat compared to control, although this trend was not significant (Supplementary Fig. S7, S8, and attached data file). Several other markers more loosely associated with A1-like phenotypes in rodent cells were found to be expressed at high levels in both control and treated human astrocytes, including TAPBP, B2M, CD44, and TIMP1 (Fig. 2, Supplementary Fig. S7), with only a slight increase in TIC-treated cells [8]. Both TAPBP and B2M are associated with Class I MHC-mediated antigen presentation system, CD44 is associated with cell adhesion, and TIMP1 serves as a metalloproteinase [8, 62–64]. Given the variable nature of astrocyte phenotypes, this may suggest that some of these markers may better serve as pan-reactive astrocyte markers, as some researchers have suggested [11, 65].

The expression of 762 genes was significantly altered in primary human fetal astrocytes treated with TIC daily for 4 days (Fig. 1A, B). Some of the most substantially down-regulated genes encoded novel lncRNA transcripts or proteins that are not currently well documented in the literature [56, 57] (Fig. 1B). One of the most significantly upregulated genes include MTFR1 (mitochondrial fission regulator 1), which is known to protect against oxidative stress, but is also associated with age-associated cognitive decline [66]. IRF1 (interferon regulatory factor 1) was also significantly upregulated, a finding that is relevant to neuroinflammatory responses given its role in inflammasome activation and regulated cell death [67]. After 6 days of daily exposure to simulated microglia signaling, the expression of 682 genes was significantly changed (Fig. 1C, D). One example is RIPK3 (receptor-interacting serine/threonine protein kinases 3), which contributes to necroptosis during inflammation, including during viral infection [68]. A surprising find was the significant upregulation of JCHAIN, which encodes the joining (J) chain component that is known for regulating polymerization of multimeric IgM and IgA. Recent evidence suggested its expression was not limited to plasma cells, but has also been characterized in dendritic cells, and it may in fact be evolutionarily conserved as a distant relative of the CXCL chemokine family [69, 70].

Gene ontological analyses of differentially expressed genes revealed which pathways were most associated with astrocyte responses to simulated microglia signaling (Table 1). The most upregulated pathways were associated with inflammation, including the NF-κB pathway thought to be responsible for the polarization of astrocytes to an A1-like phenotype [2]. Genes associated with A2-like polarization or generalized activation (pan-reactive) were not consistently upregulated in primary human fetal astrocytes exposed to simulated microglia signaling; however, markers associated with neurotoxic inflammatory responses, such as C3, NFKB1, CXCL10, CXCL1, CXCL3, LCN2, SRGN, and SLC39A14, were, further indicating polarization to an A1-like phenotype (Fig. 2, Supplementary Fig. S6).

Previous literature has shown that upregulation of the protein C3 is a well-characterized marker of A1-like astrocyte polarization [2, 9, 10]. C3 is a complement component important for coordinating the activation of the broader complement system as part of the inflammatory response. Intact intracellular C3 (~ 195 kDa) is comprised of an α-chain (~ 120 kDa) and a β-chain (~ 75 kDa) [58, 60, 71]. In both the classical and alternative complement pathways, C3 convertase cleaves C3 into several fragments with different roles in mediating local inflammation [72]. These isoforms include C3a (~ 9 kDa), an anaphylatoxin and immune cell activator, C3b (~ 185 kDa), an opsonin which can bind to cell surfaces or immune aggregates to promote phagocytosis, and its activation-associated degradation product iC3b (~ 183 kDa), which itself contributes to pathogen clearance [60]. iC3b is typically cleaved in C3c (~ 140 kDa) and C3dg (~ 42 kDa), the latter of which facilitates and amplifies B cell activation [71, 72]. An α-chain fragment of iC3b was also observed (~ 65 kDa). Elevated amounts of C3 cleavage products are thought to be an indication of continuous activation of the complement system [73]. Downstream of C3 processing, C5 cleavage by a C3b-bound C3 convertase produces a C5a fragment that activates T cells and antigen-presenting cells, as well as a C5b fragment that initiates membrane attack complex formation [72].

We observed that unstimulated human fetal astrocytes constitutively express low levels of intact intracellular C3. This was consistent with previous research that has identified endosomal stores of un-cleaved C3 to be beneficial to cell survival, permitting a rapid signaling response to external changes [74]. C3 expression significantly increased following treatment with simulated microglia signaling, consistent with conversion to an A1-like phenotype (Fig. 3A–C). The extracellular concentration of C3, which must be secreted by astrocytes to exert an effect on the local cellular environment, was significantly increased with each day of treatment (Fig. 3D). CXCL10, an important chemoattractant and another A1-like astrocyte marker, displayed an initial significant increase in secretion that ultimately returned to baseline, despite subsequent stimulation (Fig. 3E). At the RNA level, CXCL10 expression was not significantly upregulated until after 6 days of treatment had occurred (Fig. 2, Supplementary Fig. S6). In neurons, CXCL10 is stored intracellularly in vesicles [75]. This may suggest that astrocytes secrete stored CXCL10 upon activation, then upregulate its expression after an extended period of exposure to pro-inflammatory stimuli. Unfortunately, the current fetal astrocyte model cannot be taken out far enough to observe the synthesis of the new CXCL10 RNA transcripts (Supplementary Fig. S6). These alternating responses of C3 and CXCL10 may represent a function in which pro-inflammatory astrocytes first respond to inflammatory insult by initiating an early migration of other immune cells to the inflamed area via secretion of chemokines, then shift to secreting immune cell-activating complement components.

In the context of the BBB, it was unclear if BMEC-associated astrocytes could undergo an A1-like polarization response, and what effect this may have. This scenario was modeled using an in vitro BBB model comprised of primary human astrocytes seeded on the basal side of a transwell membrane and primary human BMECs seeded on the apical side. While other BBB models have used rodent cells or human cell lines, use of primary human cells may have important translational applications. Following chronic TIC exposure of the astrocyte-adjacent basal chamber, the pattern of secreted astrocyte signaling factors differed from the monoculture system. First, the transwell systems secreted C3 from co-cultured astrocytes prior to treatment exposure, in contrast to the monocultured astrocytes, indicating an early baseline level of immune activation (Fig. 6B). This may be a consequence of astrocyte association with BMECs, a distinct fetal cell type. Over time, the level of C3 present in untreated controls had substantially decreased, reaching negligible levels. This baseline level of C3 secretion was likely reached as the cells acclimated to culture. In contrast, TIC-treated cultures maintained high levels of C3 secretion, suggesting a sustained pro-inflammatory phenotype (Fig. 6B). CXCL10 secretion was significantly increased in response to treatment with simulated microglia signaling and does not begin to return to baseline control levels for several days, further suggesting an A1-like polarization response (Fig. 6C). Astrocytes associated with BMECs in the BBB model maintained high levels of chemokine secretion for longer than was observed in the monoculture system, suggesting that activated astrocytes may tailor their response in relation to their localization. In this case, astrocytes with access to the periphery via cerebral vasculature may thus display increased chemokine signaling (Fig. 3E, Fig. 6C).

No significant change in barrier integrity following consecutive daily TIC treatment occurred (Fig. 7A, C). Thus, A1-like polarization can be induced by simulated microglia signaling in BBB-associated astrocytes, but this response alone does not make a substantial impact on barrier function (Fig. 8). While loss of overall barrier integrity was not observed, increased secretion of chemokines by A1-like astrocytes may encourage transmigration of peripheral immune cells.

The importance of A1-like astrocytes in the etiology of neurodegenerative disease is well-documented, as is the polarization of astrocytes in response to pathogenic proteins; however, their role has not been explored in the context of HIV-1 infection associated with neurodegeneration, including HIV-1-associated neurocognitive disorder [2]. The viral protein HIV-1 Tat is continuously secreted by infected cells in the CNS, even when infection is well-suppressed, and Tat can elicit microglia activation [39–41]. To determine if A1-like astrocyte polarization occurs in the context of chronic HIV-1 infection, astrocytes were exposed repeatedly to the extracellular pathogenic viral protein Tat.

At the RNA level, HIV-1 Tat did not cause upregulation of A1- or A2-associated markers, suggesting that the viral protein is not able to induce A1-like polarization of astrocytes alone, at least in this experimental system (Fig. 4A–G, Supplementary Fig. S6). However, several genes did undergo significant expression changes with HIV-1 Tat exposure. One of the more highly upregulated genes was SCHIP1 (Schwannomin interacting protein 1), which encodes a protein that contributes to axon guidance in neurons [76]. Its function in astrocytes is unclear. SP7 was also significantly upregulated in primary human fetal astrocytes exposed to HIV-1 Tat; SP7 encodes a protein associated with osteocytogenesis that drives dendrite formation in maturing osteocytes and is not generally enriched in astrocytes [77]. ELAPOR2, thought to be a regulator of the bone morphogenetic protein (BMP) signaling pathway, was also upregulated [59]. The relevance of these aberrant astrocyte expression changes due to HIV-1 viral protein exposure should be investigated further.

At the protein level, western immunoblot analysis of astrocyte lysates did not detect any increase in the expression of any C3 isoforms (Fig. 5A–C). After 4 days of treatment, HIV-1 Tat failed to elicit a significant increase in secretion of C3 or CXCL10 (Fig. 5C, D). CXCL10 also did not increase following treatment with either dose of HIV-1 Tat; rather, a decrease was observed after 5 consecutive days of treatment with the high dose (Fig. 5D). This may suggest that chronic exposure to HIV-1 viral protein fails to elicit chemoattractive signal release from astrocytes that may otherwise result in increased immune infiltration and infection seeding (Fig. 8). Similarly, in the BBB model, a small increase in C3 secretion by astrocytes treated with a high dose of HIV-1 Tat was observed, though no increase in CXCL10 was observed (Supplementary Fig. S9B–C). Furthermore, no significant changes in permeability were detected (Fig. 7B). This suggests that in primary human fetal astrocytes, repeated exposure to HIV-1 Tat resulted in only slight upregulation of C3 signaling and no change in BBB integrity (Fig. 8). While we hypothesized that TIC plus HIV-1 Tat may exacerbate the A1-like phenotype, in the model tested here, we did not observe this (Supplementary Fig. S6). These results suggest that the major impact on astrocytes in relation to chronic HIV-1 infection is a consequence of microglia signaling and future experiments should investigate if astrocyte polarization arises in the presence of HIV-1-infected microglia.

In conclusion, inducing an A1-like astrocyte response using primary human astrocytes treated with simulated microglia signaling was successful. Using this model, further investigation into the role of A1-like astrocytes in various pathological conditions can be conducted. Furthermore, astrocytes associated with BMECs in an in vitro BBB model could also be stimulated to adopt an A1-like phenotype, and that this polarization did not significantly influence the functional integrity of the barrier. This model can now be used to begin to investigate A1-like astrocytes associated with CNS barriers, a topic that has not been previously explored. The final goal of these experiments was to add to the growing body of work investigating the pathologic consequences of A1-like astrocyte polarization by investigating it in the context of chronic HIV infection. The genes that were influenced by HIV-1 Tat exposure may have their own detrimental effects on astrocyte function and require further investigation. It may be possible that HIV-1 Tat elicits a unique variation of the A1-like phenotype when astrocytes are simultaneously stimulated by activated microglia signals; a response that may be further modified when in the presence of BMECs. Unlike pathogenic proteins in Alzheimer’s disease, chronic exposure to low levels of HIV-1 viral protein Tat did not independently induce astrocyte activation (Fig. 8). However, HIV-1 Tat exposure is reported to induce microglia activation, and activated microglia secrete the signals responsible for A1-like astrocyte polarization [78]. In this way, HIV-1 Tat exposure may indirectly induce A1-like astrocyte polarization in a process facilitated by activated microglia.

Interestingly, HIV-1 Tat and other viral proteins like Nef can be excreted via exosome release, although it is not yet clear what proportion of Tat in the CNS is free versus packaged in exosomes [79–82]. It is possible that astrocytes may also take up HIV-1 Tat or Nef in a similar manner. Under healthy conditions, astrocytes favor uptake of neuronal and monocyte-derived extracellular vesicles, while under pathological conditions, astrocytes are more inclined to take up extracellular vesicles released by microglia, other astrocytes, and various stem cells, and this process can promote the expression of pro-inflammatory mediators [83]. In the context of chronic HIV-1 infection, astrocytes may very well be inclined to take up Tat-containing exosomes, and this may represent a more efficient way of introducing Tat to astrocytes. The focus on the impact of HIV-1 Tat is a limitation of this work, as the effect of other neuropathogenic HIV-1 viral proteins on astrocyte phenotype warrants further investigation, including the Env glycoprotein component gp120 [84–88], the regulatory viral protein R (Vpr) [89–92], and Nef [93]. Furthermore, other neurotoxic proteins are secreted either in supernatants or extracellular vesicles by other CNS cells as an inflammatory response during HIV-1 infection [94–96]. All of these inflammatory proteins are present in the CNS during HIV-1 infection, and all of them have been linked to associated neuropathologies and HAND, making them of particular importance for future research [96, 97]. Tat has been the most widely studied, with in vitro, ex vivo, animal models, and human samples all showing links to neuropathogenesis, as well as pathogenic and behavior outcomes similar to those seen in PLWH, and expression of Tat correlating to HAND in ART-controlled individuals. However, more recent studies have demonstrated Nef expression by CNS cells, including astrocytes, is associated with neurotoxicity and NCI in PLWH [98, 99]. Future investigations should broaden the scope and include astrocyte exposure to an array of HIV-1 accessory proteins in order to best represent in vivo disease conditions. The in vitro primary human astrocyte model of A1-like astrocyte polarization verified in these experiments can be expanded on with other methodologies, such as induced pluripotent stem cell (iPSC)-derived astrocyte models. Further exploration into the effects of inflammatory astrocyte polarization on CNS barrier integrity, as well as treatment with different pathogenic proteins, may provide insights into the role of neurotoxic reactive astrocytes in neuroviral pathologies.

## Supplementary Information

Below is the link to the electronic supplementary material.

ESM 1 (PDF 204 KB)

ESM 2 (DOCX 7.22 MB)

## Data Availability

Raw data available upon request. RNA sequencing datasets generated during and/or analyzed during the current study are available in the NCBI Gene Expression Omnibus (GEO) repository. The GEO accession number is GSE291361 and can be accessed at https://www.ncbi.nlm.nih.gov/geo/query/acc.cgi?acc = GSE291361 (access token: ytwhaamwhtmzfyn).
