# Supplementary material for: Inflammatory microglia signals drive A1-like polarization of astrocytes even in the presence of HIV-1 Tat
Source: Mol Neurobiol. 2025 Dec 4;63(1):251. doi: 10.1007/s12035-025-05409-z (PMC12678590; doi:10.1007/s12035-025-05409-z)
Supplement: Supplementary file 1 — (PDF 204 KB) [file 12035_2025_5409_MOESM1_ESM.pdf]

Near-infrared western blot data portrayed in Fig. 3 and Fig. 5 were obtained from the following 3 gels.

Fig. 3A - gel #1 (Trial D) – representative blot
Fig. 3B - gel #1 (Trial D) – quantification
Fig. 3C - gel #1 (Trial D & Trial H) and gel #2 (Trial C) – fold-change

Fig. 5A - gel #3 (Trial D) – representative blot
Fig. 5B - gel #3 (Trial D) – quantification
Fig. 5C - gel #2 (Trial C) and gel #3 (Trial D) – fold-change

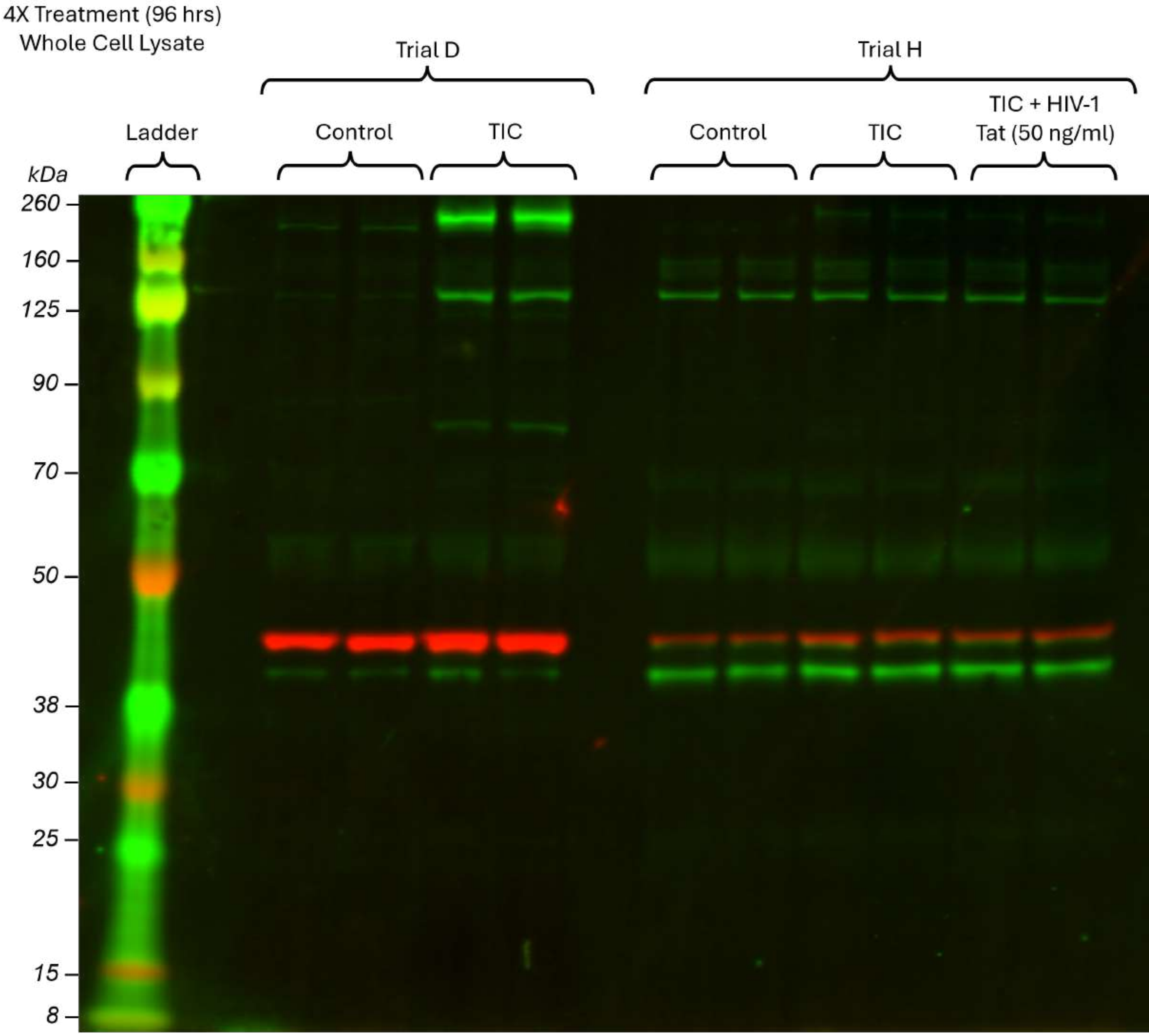
4X Treatment (96 hrs)
Whole Cell Lysate
Trial D
Trial H
Ladder
Control
TIC
Control
TIC
TIC + HIV-1
Tat (50 ng/ml)
kDa
260
160
125
90
70
50
38
30
25
15
8

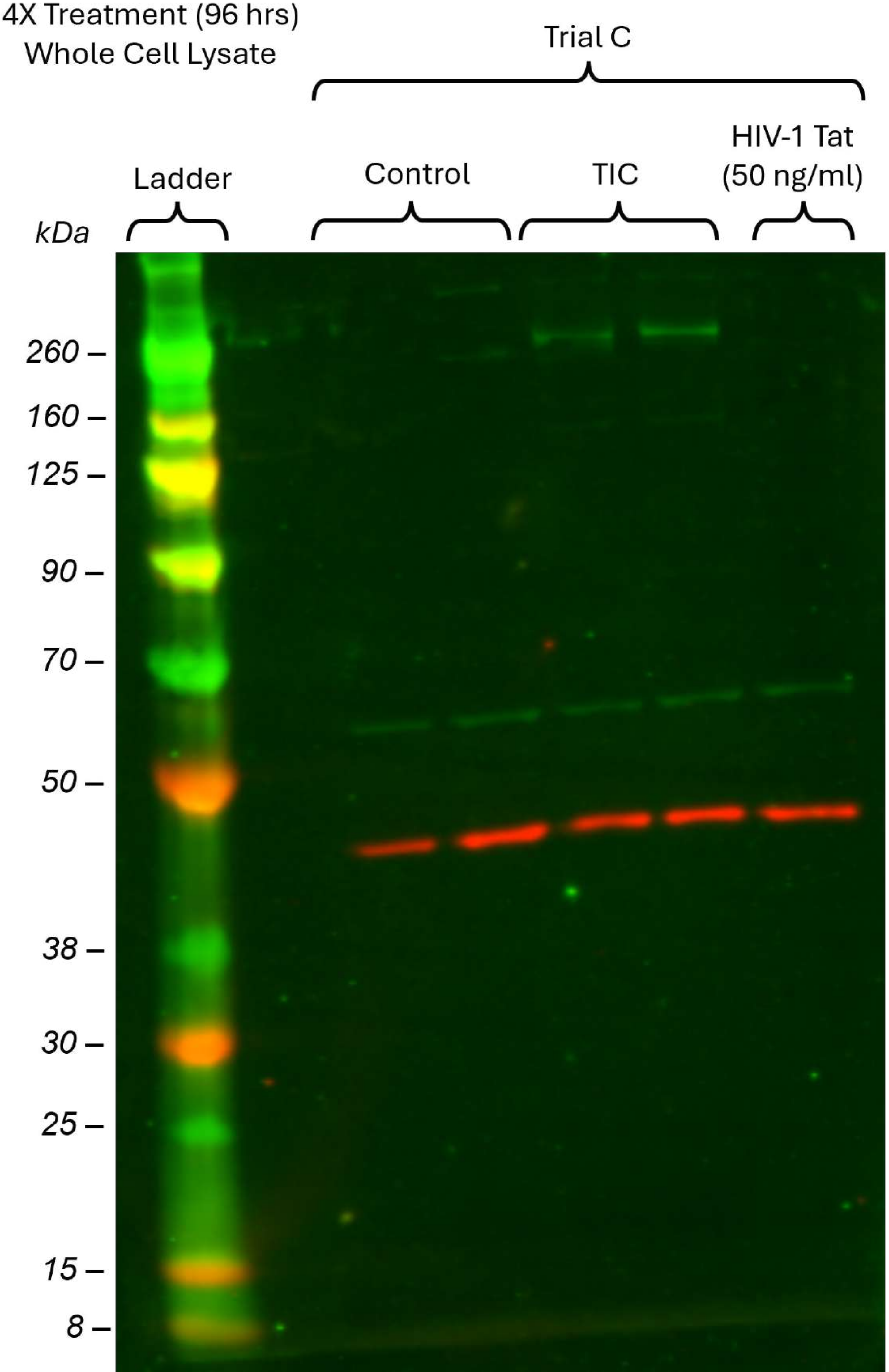
4X Treatment (96 hrs)
Whole Cell Lysate
Trial C
Ladder
Control
TIC
HIV-1 Tat
(50 ng/ml)
kDa
260
160
125
90
70
50
38
30
25
15
8

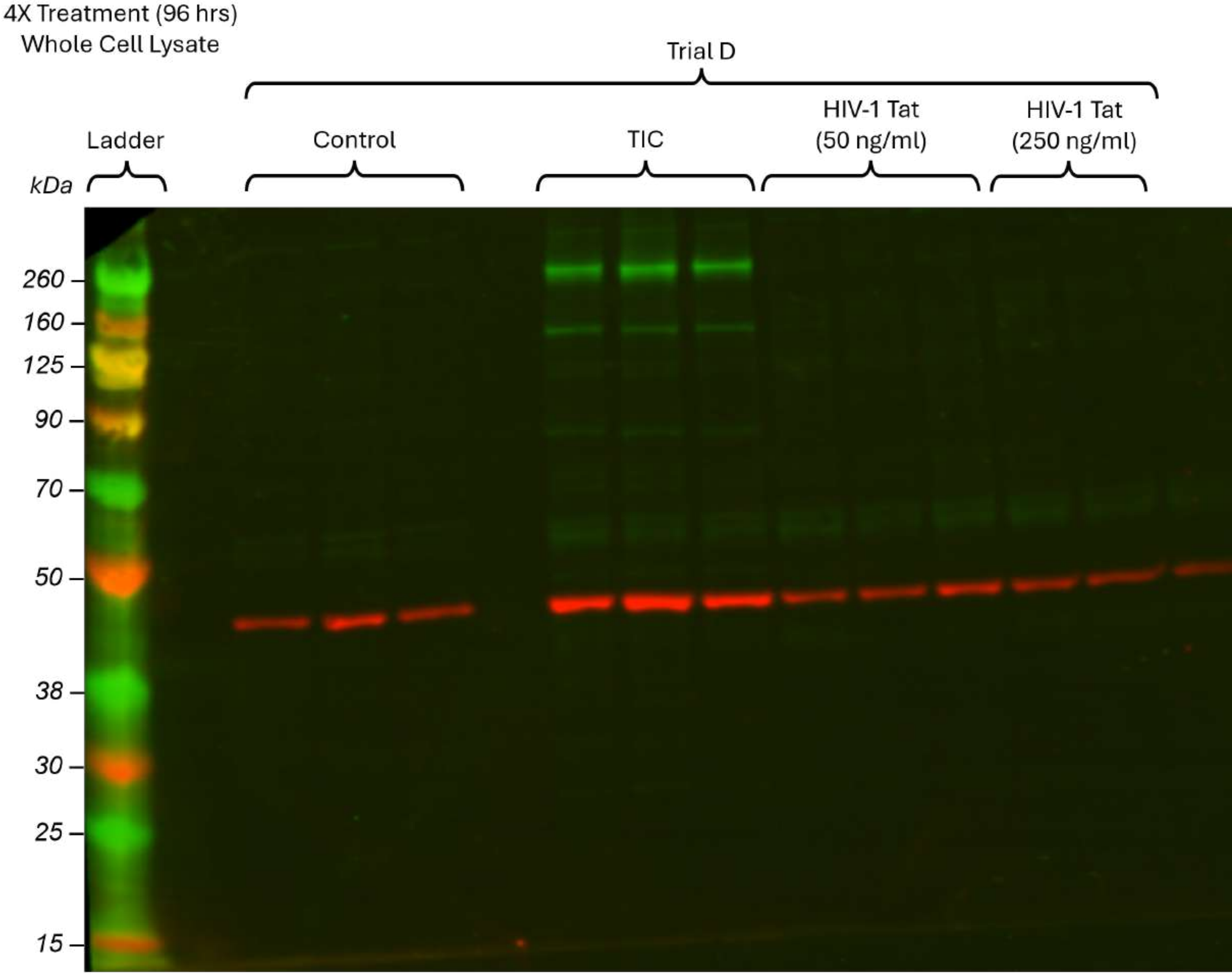
4X Treatment (96 hrs)
Whole Cell Lysate
Trial D
Ladder
Control
TIC
HIV-1 Tat
(50 ng/ml)
HIV-1 Tat
(250 ng/ml)
kDa
260
160
125
90
70
50
38
30
25
15
