# Supplementary material for: Inflammatory microglia signals drive A1-like polarization of astrocytes even in the presence of HIV-1 Tat
Source: Mol Neurobiol. 2025 Dec 4;63(1):251. doi: 10.1007/s12035-025-05409-z (PMC12678590; doi:10.1007/s12035-025-05409-z)
Supplement: Supplementary file 2 — (DOCX 7.22 MB) [file 12035_2025_5409_MOESM2_ESM.docx]

**Inflammatory microglia signals drive A1-like polarization of astrocytes even in the presence of HIV-1 Tat**

Jill M. Lawrence^1,2,3^, Will Dampier^2^, Joshua Chang Mell^2,4,5,6^, Diehl R. De Souza^1,2,3^, Kayla Schardien^1,7^, Kyle Yeakle^1,8^, R. Jordan Barnett^2,9^, Bhaswati Sen^2,4,5,6^, Azad Ahmed^2,5,6^, Michael Bouchard^8^, Brian Wigdahl^2,3,10^, and Michael R. Nonnemacher^2,3,10^

**SUPPLEMENTARY INFORMATION**


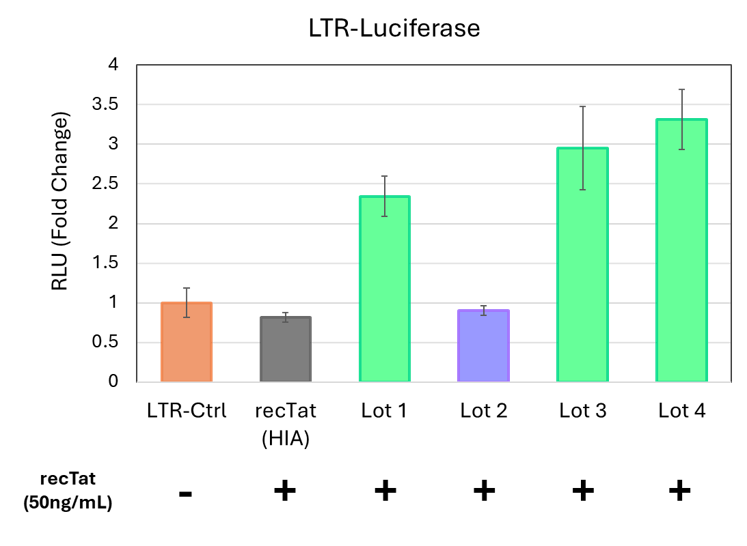


**Supplementary Fig. S1** Exogenous full-length HIV-1 Tat is functionally active. Hepatocytes were transfected with pGL3-HIV1-LTR-luciferase and treated with different lots of 50ng/mL of HIV1 Tat or heat-inactivated (HIA) HIV1 Tat. Primary rat hepatocytes were collected 12 hours post-treatment and processed for a luciferase assay to assess transactivation of the HIV1 LTR by purified Tat. Purified HIV1 Tat from all lots except one transactivated the HIV1 LTR, demonstrating that purified HIV1 Tat is functionally active. The inactive lot of purified HIV-1 Tat was not used in further experimentation. RLU = relative light units; LTR = long terminal repeat; HIA = heat-inactivated; Ctrl = control


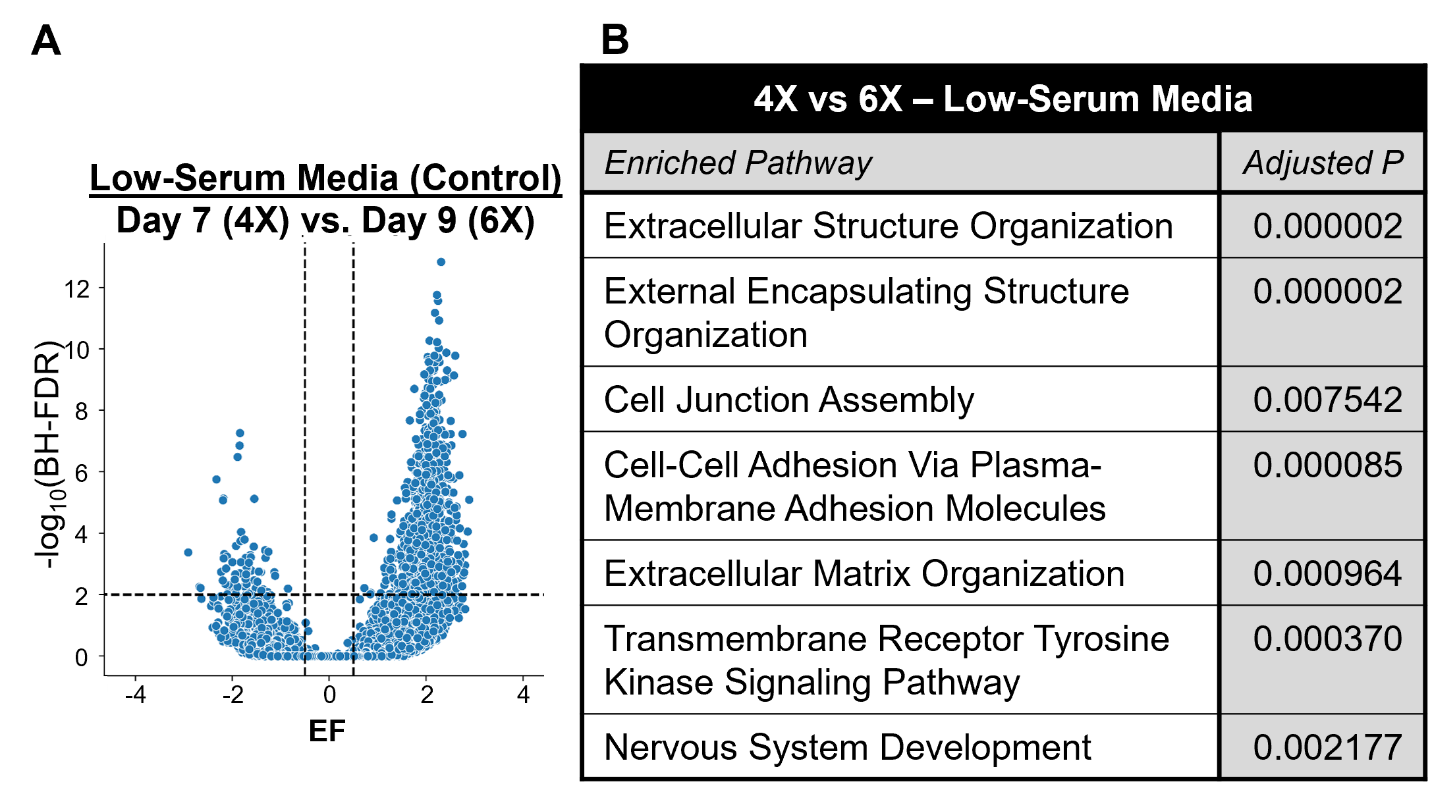


**Supplementary Fig. S2** Gene expression changes in untreated (control) primary human fetal astrocytes between culture day 7 and day 9. **(A)** Volcano plot depicting the degree of downregulation and upregulation of different genes after 6 days (culture day 9) of the control condition of low-serum media replacement compared to 4 days (culture day 7). **(B)** Gene enrichment pathway analysis of the transcripts with the most significant expression upregulation in astrocytes on culture day 9 (6X) compared to day 7 (4X). BH-FDR = Benjamini-Hochberg False Discovery Rate; EF = Effect Size; N=3 (per condition)

**Supplementary Fig. S3** Repeated exposure of primary human fetal astrocytes to TNF-α, IL-1α, and C1q elicits a greater increase in expression of A1-associated C3 cleavage products compared to single exposures. Mean log_2_ fold change of intact C3 expression in treated cells compared to time-matched control was calculated using the mean relative fluorescence intensity of protein bands quantified from NIR western immunoblots, with band intensity values corrected and normalized to β-actin and normalized to β-actin. 1X represented by blue stripes (n=3), 3X represented by blue diamond check pattern (n=3), and 4X represented by blue with white spots (n=7) Error bars represent SD.


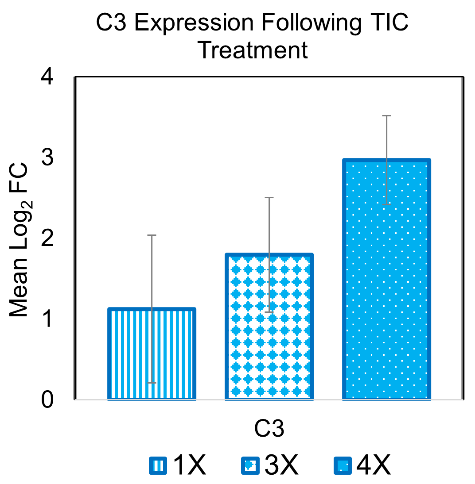


**Supplementary Table. 1** Genetic markers of different astrocyte phenotypes.

| **Astrocyte Phenotype Markers** | | | | |
| --- | --- | --- | --- | --- |
| *Phenotype* | *Gene* | *Function* | *Citation* |  |
| **Astrocyte** | **GFAP** | Cytoskeletal intermediate filament | Liddelow, et al. 2017 [9] |  |
| **Astrocyte** | **S100B** | Ca^2+^-binding protein | Liddelow, et al. 2017 [9] |  |
| **A1-like** | **C3** | Complement component | Liddelow, et al. 2017 [9] |  |
| **A1-like** | **C1S** | Complement component | Zamanian, et al. 2012 [8] |  |
| **A1-like** | **NFKB1** | Inflammatory transcription factor | Huat, et al. 2024 [100] |  |
| **A1-like** | **RELA** | Mediates response to cytokines | Diehl, et al. 1995 [101] |  |
| **A1-like** | **TNF** | Inflammatory cytokine | Fróes, et al. 2024 [102] |  |
| **A1-like** | **CXCL10** | Chemokine (T cells and NK cells) | Clarke, et al. 2018 [11] |  |
| **A1-like** | **CXCL1** | Chemokine (neutrophils) | Lu, et al. 2005 [103] |  |
| **A1-like** | **CXCL3** | Chemokine (monocytes) | Lu, et al. 2005 [103] |  |
| **A1-like** | **LCN2** | Iron sequestration, innate immunity | Wang, et al. 2019 [24] |  |
| **A1-like** | **SLC39A14** | Iron uptake during inflammation | Routhe, et al. 2020 [104] |  |
| **A1-like** | **TAPBP** | Mediates MHC class I interactions | Zamanian, et al. 2012 [8] |  |
| **A1-like** | **GBP3** | IFN-γ-induced GTPase | Ugalde, et al. 2020 [105] |  |
| **A1-like** | **B2M** | MHC class I component | Zamanian, et al. 2012 [8] |  |
| **A1-like** | **SRGN** | Inflammatory proteoglycan | Klemens, et al. 2019 [25] |  |
| **A1-like** | **GBP2** | IFN-γ-induced GTPase | Dickinson, et al. 2023 [106] |  |
| **A1-like** | **PSMB8** | Immunoproteasome subunit | Nguyen, et al. 2023 [107] |  |
| **A1-like** | **SERPINA3** | Peptidase inhibitor | Masvekar, et al. 2020 [23] |  |
| **A1-like** | **IER3** | Protects cell from apoptosis | Zamanian, et al. 2012 [8] |  |
| **A1-like** | **AMIGO2** | Regulates immune cell function | Fang, et al. 2022 [108] |  |
| **A1-like** | **SLC22A4** | Cation transport | Zamanian, et al. 2012 [8] |  |
| **A1-like** | **CLIC1** | Cl^-^ channel | Masvekar, et al. 2020 [23] |  |
| **A1-like** | **S1PR3** | Immune cell regulation | Zamanian, et al. 2012 [8] |  |
| **A1-like** | **CRISPLD2** | Associated with sepsis | Zamanian, et al. 2012 [8] |  |
| **A1-like** | **CD44** | Adhesion, lymphocyte activation | Bradford, et al. 2019 [63] |  |
| **A1-like** | **SERPING1** | Serine protease inhibitor | Fang, et al. 2022 [108] |  |
| **A1-like** | **FKBP5** | Glucocorticoid receptor modulation | Clarke, et al. 2018 [11] |  |
| **A-like** | **TIMP1** | Metalloproteinase regulation | Barbar, et al. 2020 [64] |  |
| **A1-like** | **STAT3** | Transcription factor | Zhang, et al. 2020 [109] |  |
| **A2-like** | **S100A10** | Ca^2+^-binding protein | King, et al. 2020 [110] |  |
| **A2-like** | **TGFB1** | Growth factor | Xu, et al. 2018 [18] |  |
| **A2-like** | **BDNF** | Neurotrophic factor | Wang, et al. 2019 [24] |  |

**Supplementary Fig. S4** Chronic exposure of primary human astrocytes to simulated microglia signaling, but not HIV-1 Tat, induces increased A1-associated Lipocalin2 secretion. **(A)** ELISA measuring lipocalin2 (pg/ml) secretion in monocultured astrocyte-conditioned media over the course of 4 days of treatment with TIC. **(B)** Lipocalin2 (pg/ml) secretion in monocultured astrocyte-conditioned media across 4 days of treatment with HIV-1 Tat (50 ng/ml) and TIC + HIV-1 Tat (50 ng/ml) combined. **(C)** ELISA measuring lipocalin2 (pg/ml) secretion in astrocyte-conditioned media harvested from the astrocyte-BMEC co-culture transwell system throughout repeated treatments with TIC. **(D)** Lipocalin2 (pg/ml) secretion in cocultured astrocyte-conditioned media from a BBB model across 4 days of treatment with HIV-1 Tat (50 ng/ml) and TIC + HIV-1 Tat (50 ng/ml) combined. OD measured in duplicate at 450 nm and corrected for negative values within each set of assay results. Each data point represents an n=2. Control values are represented by orange lines, TIC-treated sample values represented by blue diamonds, HIV-1 Tat (50 ng/ml) treatment is represented by green triangles, and TIC + HIV-1 Tat (50 ng/ml) treatment is represented by yellow circles. Statistical significance determined using student’s t-test comparing time-matched untreated to treated corrected mean values. Error bars represent SD. *p≤0.05, **p≤0.005, ***p≤0.0005


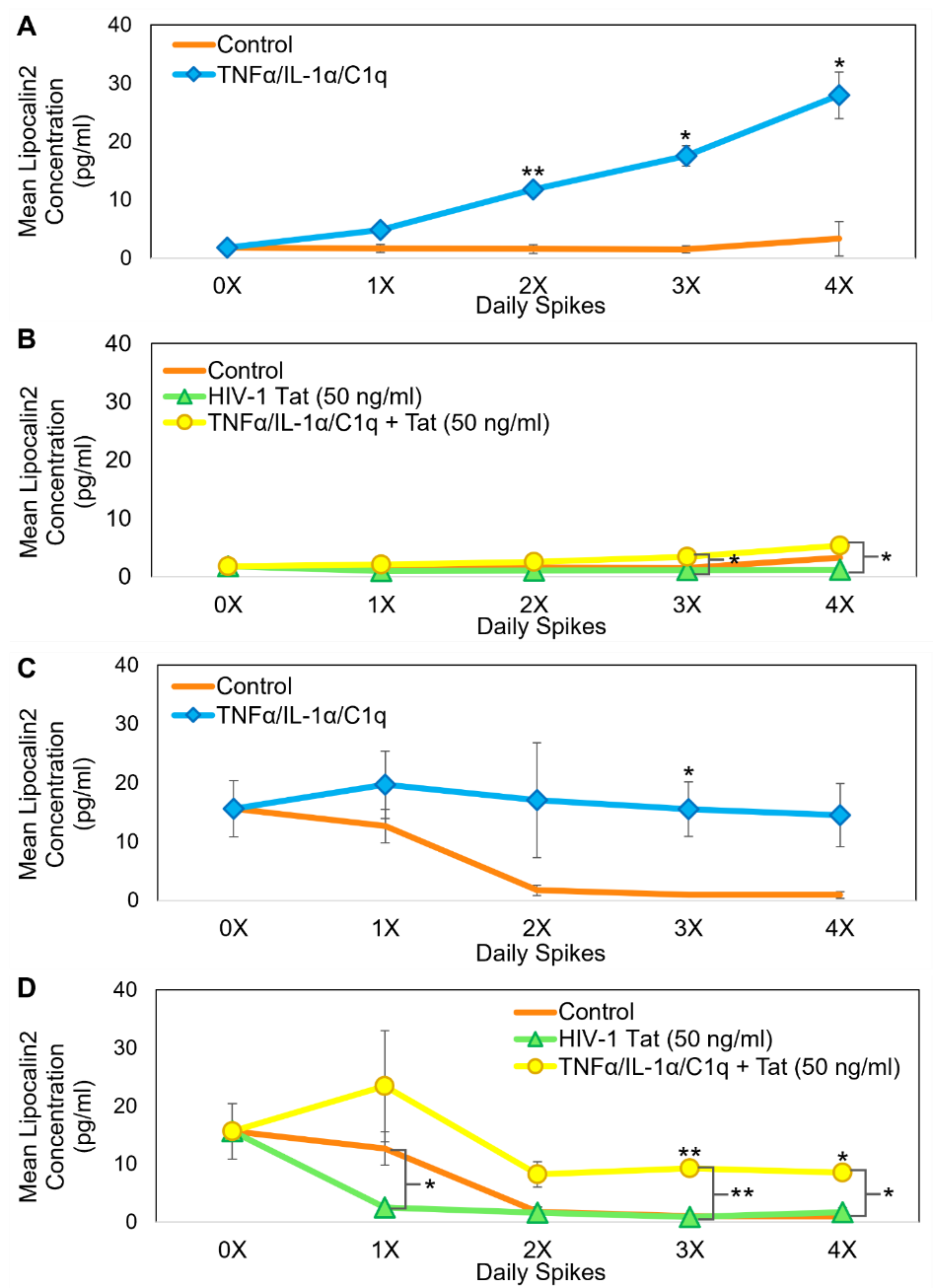

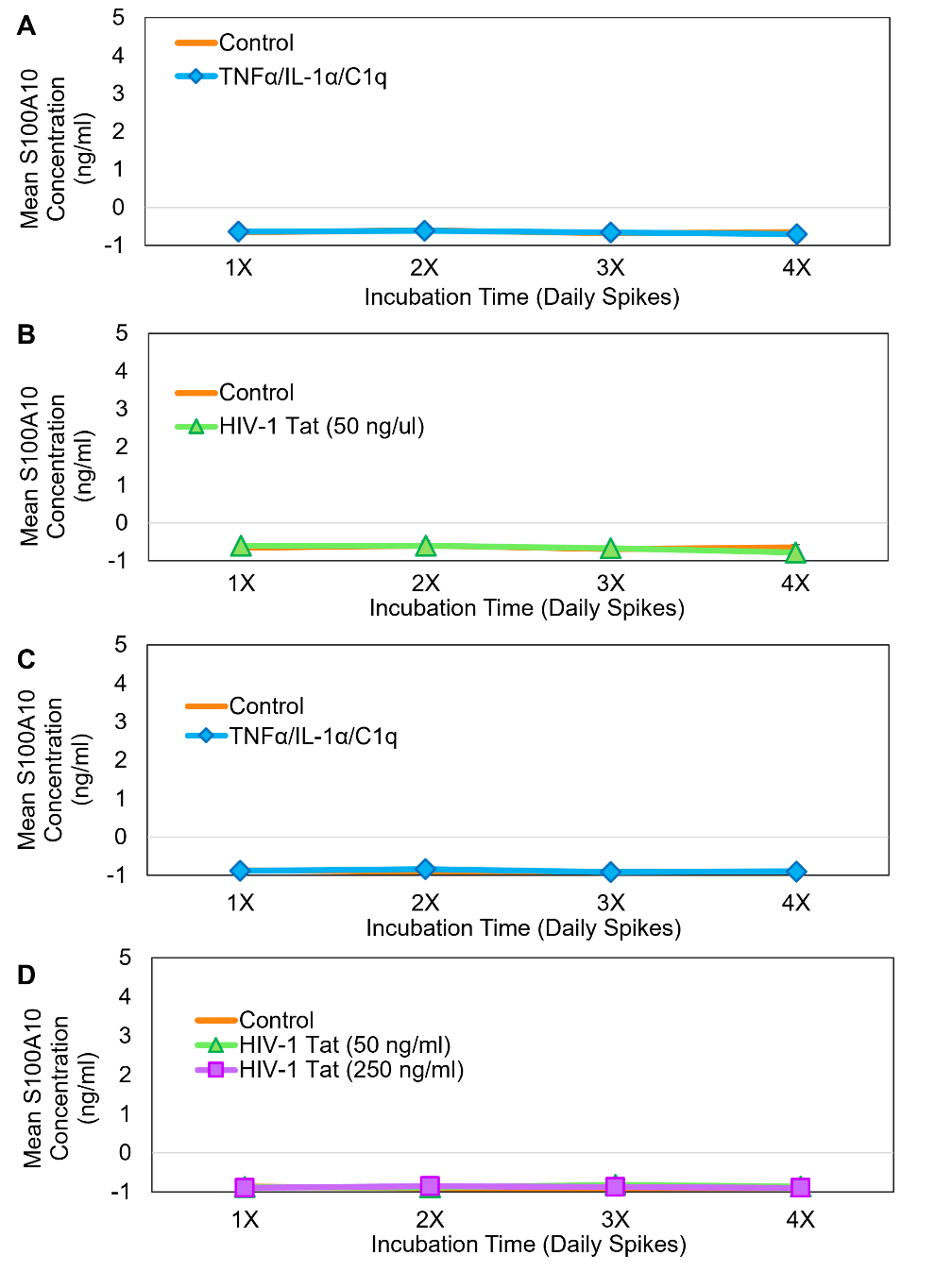


**Supplementary Fig. S5** Chronic exposure of primary human astrocytes to simulated microglia signaling or HIV-1 Tat fails to induce increased A2-associated S100A10 secretion. **(A)** ELISA measuring S100A10 (ng/ml) secretion in monocultured astrocyte-conditioned media over the course of 4 days of treatment with TIC. **(B)** S100A10 secretion in monocultured astrocyte-conditioned media across 4 days of treatment with HIV-1 Tat (50 ng/ml). **(C)** ELISA measuring S100A10 in astrocyte-conditioned media harvested from the astrocyte-BMEC co-culture transwell system throughout repeated treatments with TIC. **(D)** S100A10 secretion in cocultured astrocyte-conditioned media from a BBB model across 4 days of treatment with HIV-1 Tat (50 ng/ml) or HIV-1 Tat (250 ng/ml). OD measured in duplicate at 450 nm and corrected for negative values within each set of assay results. Each data point represents an n=2. Control values are represented by orange lines, TIC-treated sample values represented by blue diamonds, HIV-1 Tat (50 ng/ml) treatment is represented by green triangles, and HIV-1 Tat (250 ng/ml) treatment is represented by purple squares. Statistical significance determined using student’s t-test comparing time-matched untreated to treated corrected mean values. Error bars represent SD. *p≤0.05, **p≤0.005, ***p≤0.0005


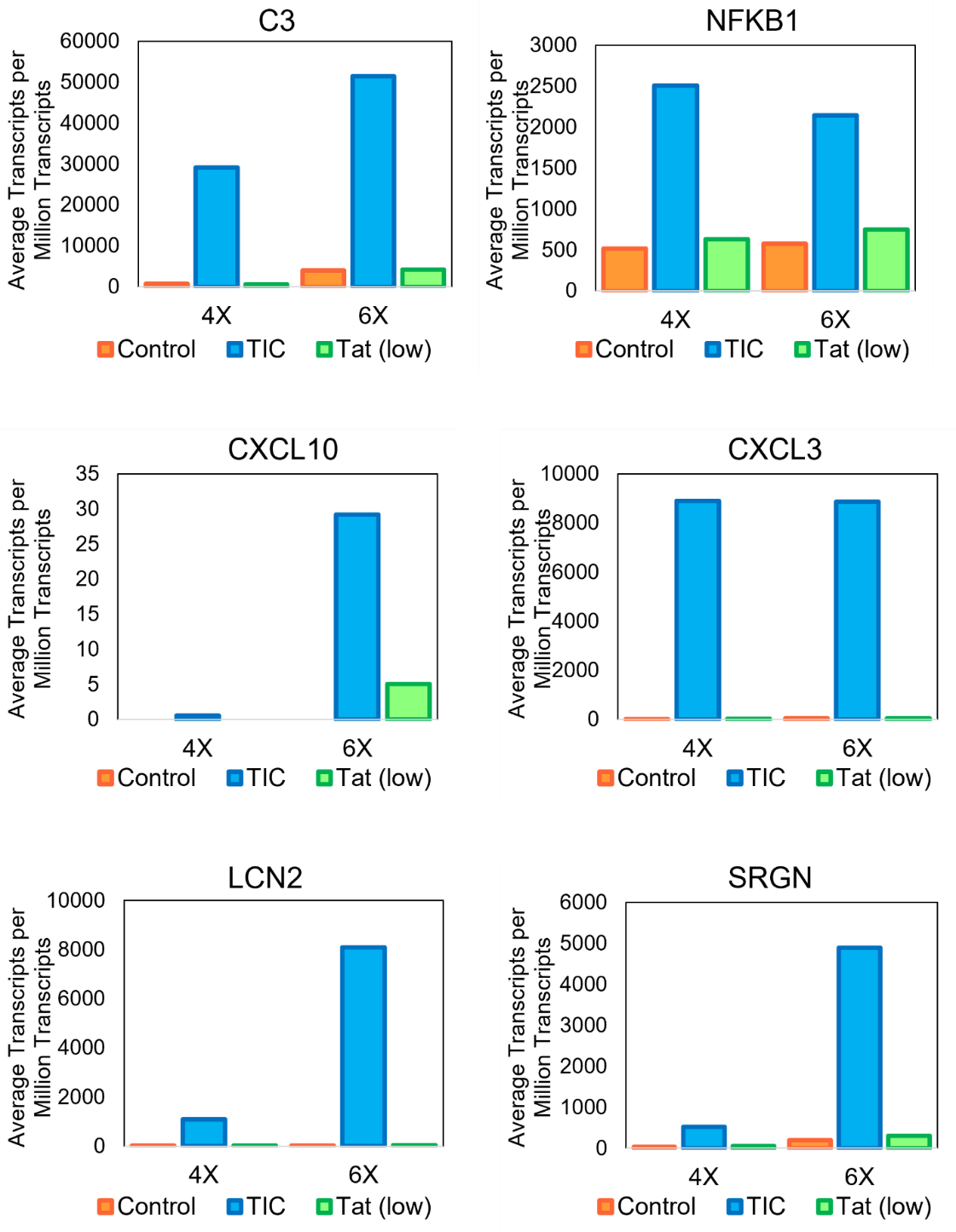


**Supplementary Fig. S6** Expression of common astrocyte phenotype-associated genetic markers is consistent with TIC-induced A1-like polarization. Highlighted markers of A1-like astrocyte phenotype in response to prolonged TIC treatment and treatment with HIV-1 Tat (50 ng/ml) after 4 and 6 days of treatment. A1-associated markers include C3, NFKB1, CXCL10, CXCL3, LCN2, and SRGN. Average expression values (transcripts per million transcripts) (n=3).

**Supplementary Fig. S7** Expression of different astrocyte phenotype-associated and HIV-1 Tat exposure-associated markers across all treatment conditions. Heatmap depicting expression level for common markers of pan-reactive, A1-assocaited, and A2-associated genes, as well as genes found to be differentially regulated by exposure to HIV-1 Tat. Values were z-scaled by gene with blue representing expression below average and magenta indicating above average expression. Each column represents the average expression from 3 samples.


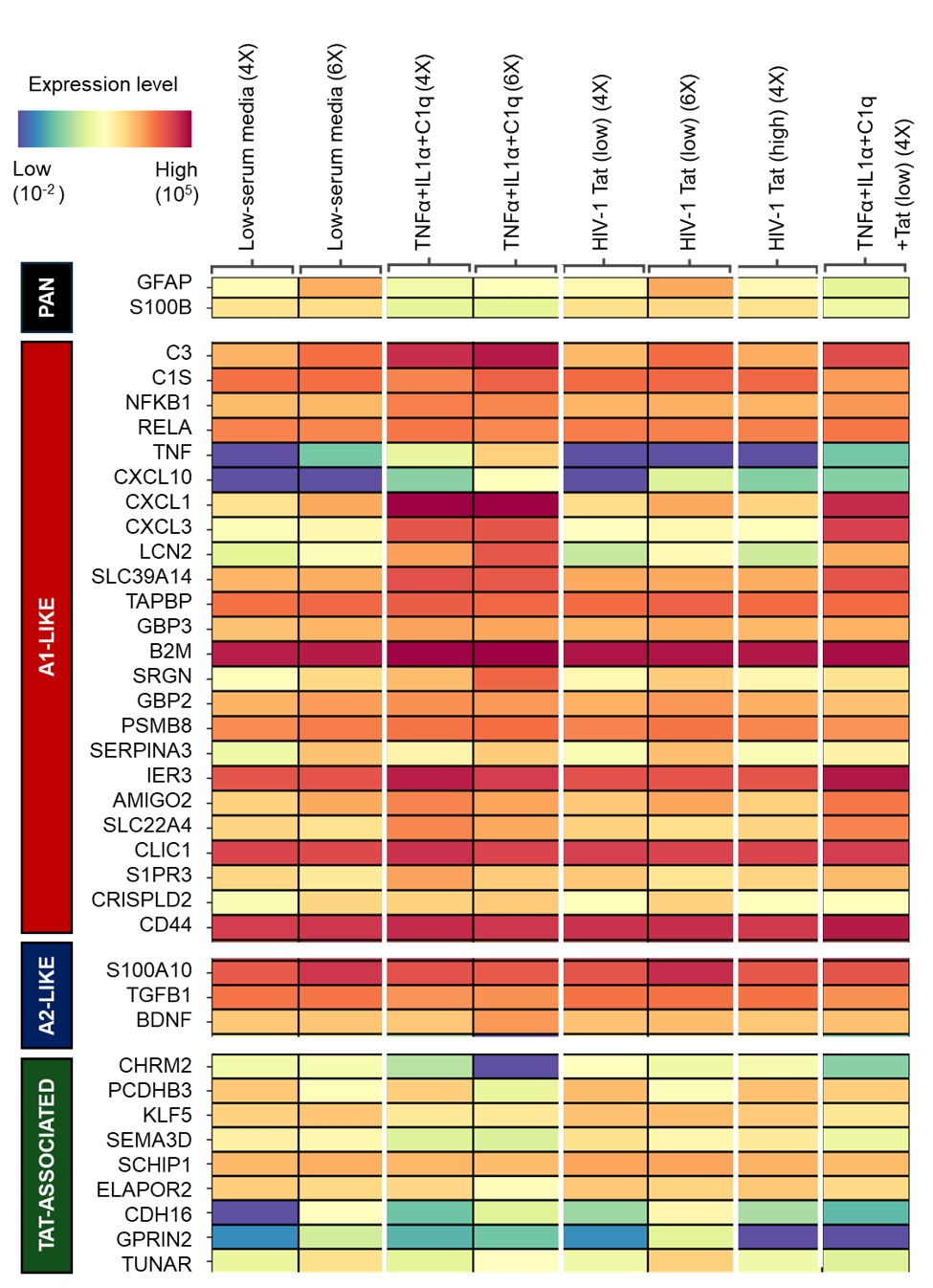


**Supplementary Fig. S8** Change in expression standard deviation of different astrocyte phenotype-associated and HIV-1 Tat exposure-associated markers across all treatment conditions. Heatmap depicting expression level for common markers of pan-reactive, A1-assocaited, and A2-associated genes, as well as genes found to be differentially regulated by exposure to HIV-1 Tat. Expression levels are depicted by change in standard deviation (-3 to 3). Each column represents the average expression from 3 samples.


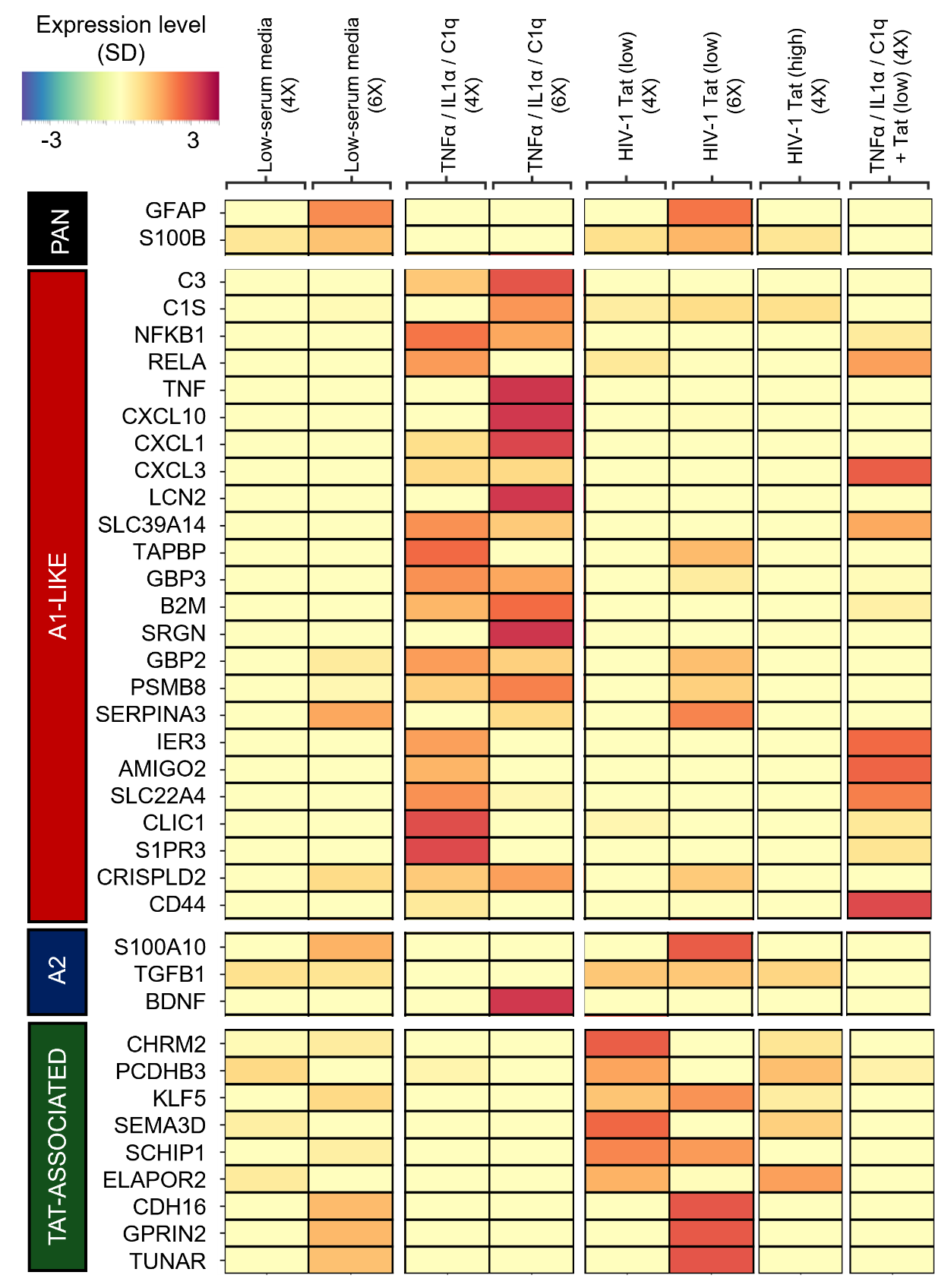


**
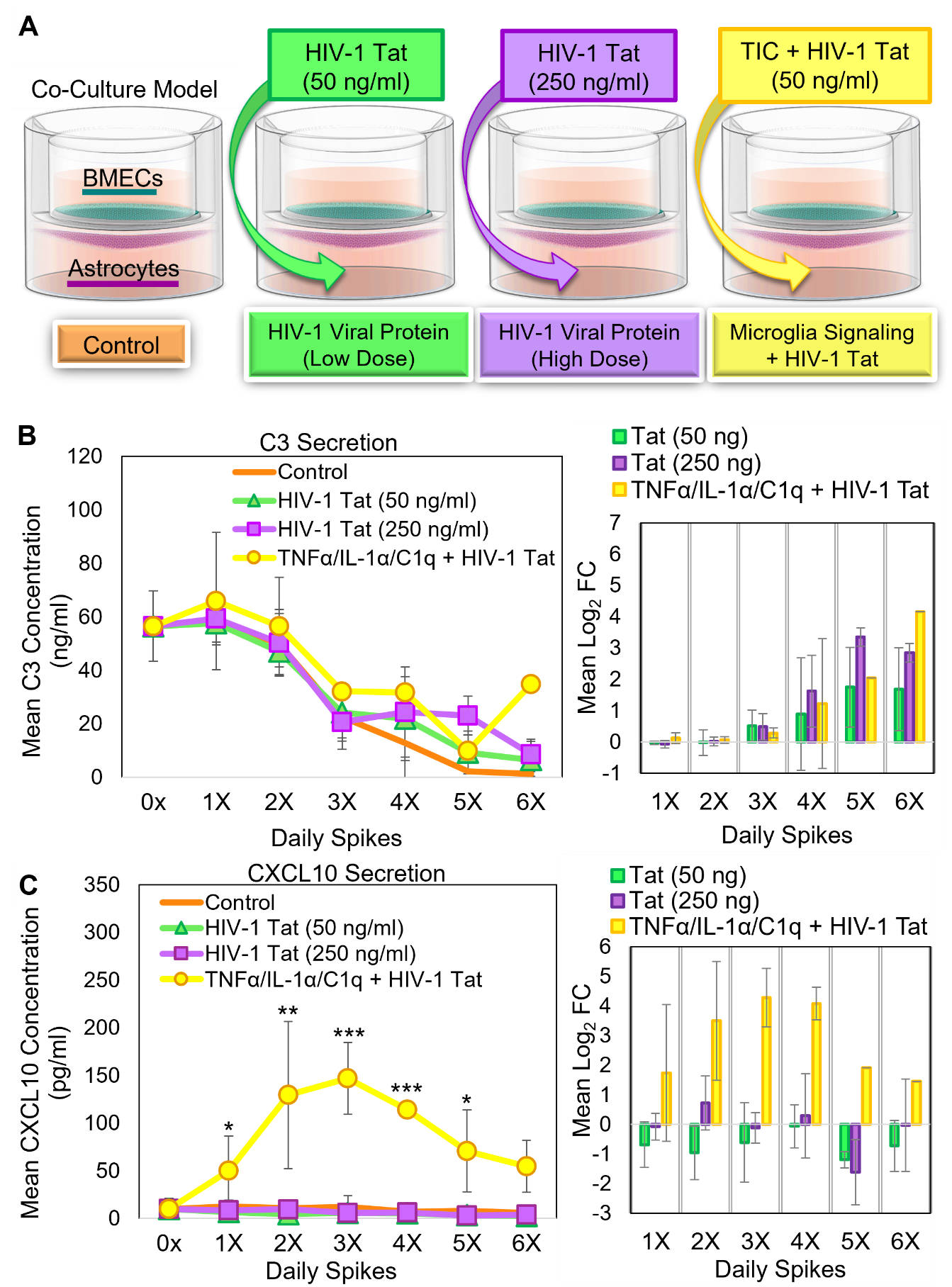
**

**Supplementary Fig. S9** Primary human astrocytes co-cultured with BMECs in an in vitro BBB model did not significantly upregulate secretion of A1-associated pro-inflammatory signals following exposure to HIV-1 Tat. **(A)** Diagram of transwell co-culture system. Transwell inserts are seeded with astrocytes and BMECs on day 0. Treatment with low-serum media (control), HIV-1 Tat (50 ng/ml), HIV-1 Tat (250 ng/ml), or TIC and HIV-1 Tat (50 ng/ml) occurred every 24 hours for 6 days. Astrocyte-conditioned media was harvested each day prior to daily spike and ELISA analysis was performed to determine expression profiles of A1-associated pro-inflammatory signals. **(B)** ELISA measuring C3 (ng/ml) and mean log_2_ fold change in astrocyte-conditioned media harvested each day prior. **(C)** ELISA measuring CXCL10 concentration (pg/ml) and mean log_2_ fold change in astrocyte-conditioned media in the co-culture model. OD measured in duplicate at 450 nm and corrected for negative values within each set of assay results. Plotted is the mean of experimental replicate averages. In panels D and E, the 0X timepoint is represented by an experimental n=5 and technical n=16. Control values are represented by orange lines (see Fig. 6). HIV-1 Tat (50 ng/ml) was represented by green triangles (1X: experimental n=5, technical replicate n=10; 2X: experimental n=5, technical replicate n=10; 3X: experimental n=5, technical replicate n=10; 4X: experimental n=5, technical replicate n=11; 5X: experimental n=3, technical replicate n=8; 6X: experimental n=3, technical replicate n=7). HIV-1 Tat (250 ng/ml) was represented by purple squares (1X: experimental n=4, technical replicate n=9; 2X: experimental n=4, technical replicate n=9; 3X: experimental n=4, technical replicate n=9; 4X: experimental n=4, technical replicate n=9; 5X: experimental n=3, technical replicate n=8; 6X: experimental n=3, technical replicate n=7). Samples treated with TIC + HIV-1 Tat (50 ng/ml) are represented by yellow circles. (1X: experimental n=3, technical replicate n=8; 2X: experimental n=3, technical replicate n=8; 3X: experimental n=3, technical replicate n=8; 4X: experimental n=3, technical replicate n=8; 5X: experimental n=2, technical replicate n=6; 6X: experimental n=2, technical replicate n=5). For all assays, statistical significance determined using student’s t-test comparing untreated to treated corrected mean values. Error bars represent SD. *p≤0.05, **p≤0.005, ***p≤0.0005

**References**

100. Jong Huat T, Camats-Perna J, Newcombe EA, Onraet T, Campbell D, Sucic JT, Martini A, Forner S, et al. (2024) The impact of astrocytic NF-κB on healthy and Alzheimer’s disease brains. Sci Rep 14:14305. https://doi.org/10.1038/s41598-024-65248-1

101. Diehl JA, Tong W, Sun G, Hannink M (1995) Tumor necrosis factor-alpha-dependent activation of a RelA homodimer in astrocytes. Increased phosphorylation of RelA and MAD-3 precede activation of RelA. J Biol Chem 270:2703–2707. https://doi.org/10.1074/jbc.270.6.2703

102. Fróes FT, Da Ré C, Taday J, Galland F, Gonçalves CA, Leite MC (2024) Palmitic acid, but not other long-chain saturated fatty acids, increases S100B protein and TNF-α secretion by astrocytes. Nutr Res 122:101–112. https://doi.org/10.1016/j.nutres.2023.12.007

103. Lu W, Maheshwari A, Misiuta I, Fox SE, Chen N, Zigova T, Christensen RD, Calhoun DA (2005) Neutrophil-specific chemokines are produced by astrocytic cells but not by neuronal cells. Brain Res Dev Brain Res 155:127–134. https://doi.org/10.1016/j.devbrainres.2005.01.004

104. Routhe LJ, Andersen IK, Hauerslev LV, Issa II, Moos T, Thomsen MS (2020) Astrocytic expression of ZIP14 (SLC39A14) is part of the inflammatory reaction in chronic neurodegeneration with iron overload. Glia 68:1810–1823. https://doi.org/10.1002/glia.23806

105. Ugalde CL, Lewis V, Stehmann C, McLean CA, Lawson VA, Collins SJ, Hill AF (2020) Markers of A1 astrocytes stratify to molecular sub-types in sporadic Creutzfeldt-Jakob disease brain. Brain Commun. https://doi.org/10.1093/braincomms/fcaa029

106. Dickinson MS, Kutsch M, Sistemich L, Hernandez D, Piro AS, Needham D, Lesser CF, Herrmann C, et al. (2023) LPSaggregating proteins GBP1 and GBP2 are each sufficient to enhance caspase-4 activation both in cellulo and in vitro. Proc Natl Acad Sci U S A 120:e2216028120. https://doi.org/10.1073/pnas.2216028120

107. Nguyen HD, Kim YE, Nhat Nguyen LT, Kwak IH, Lee YK, Kim YJ, Hai Nguyen TT, Pham HN, et al. (2023) Upregulation of immunoproteasome PSMB8 is associated with Parkinson’s disease. Parkinsonism Relat Disord 114:105797. https://doi.org/10.1016/j.parkreldis.2023.105797

108. Fang Y, Ding X, Zhang Y, Cai L, Ge Y, Ma K, Xu R, Li S, et al. (2022) Fluoxetine inhibited the activation of A1 reactive astrocyte in a mouse model of major depressive disorder through astrocytic 5-HT(2B)R/β-arrestin2 pathway. J Neuroinflammation 19:23. https://doi.org/10.1186/s12974-022-02389-y

109. Zhang HY, Wang Y, He Y, Wang T, Huang XH, Zhao CM, Zhang L, Li SW, et al. (2020) A1 astrocytes contribute to murine depression-like behavior and cognitive dysfunction, which can be alleviated by IL-10 or fluorocitrate treatment. J Neuroinflammation 17:200. https://doi.org/10.1186/s12974-020-01871-9

110. King A, Szekely B, Calapkulu E, Ali H, Rios F, Jones S, Troakes C (2020) The increased densities, but different distributions, of both C3 and S100A10 immunopositive astrocyte-like cells in Alzheimer’s disease brains suggest possible roles for both A1 and A2 astrocytes in the disease pathogenesis. Brain Sci. https://doi.org/10.3390/brainsci10080503
